# Production of ricinoleic acid-containing monoestolide triacylglycerides in an oleaginous diatom, *Chaetoceros gracilis*

**DOI:** 10.1038/srep36809

**Published:** 2016-11-10

**Authors:** Masataka Kajikawa, Tatsuki Abe, Kentaro Ifuku, Ken-ichi Furutani, Dongyi Yan, Tomoyo Okuda, Akinori Ando, Shigenobu Kishino, Jun Ogawa, Hideya Fukuzawa

**Affiliations:** 1 Graduate School of Biostudies, Kyoto University, Kyoto 606-8502, Japan; 2 Graduate School of Agriculture, Kyoto University, Kyoto 606-8502, Japan

## Abstract

Ricinoleic acid (RA), a hydroxyl fatty acid, is suitable for medical and industrial uses and is produced in high-oil-accumulating organisms such as castor bean and the ergot fungus *Claviceps*. We report here the efficient production of RA in a transgenic diatom *Chaetoceros gracilis* expressing the fatty acid hydroxylase gene (*CpFAH*) from *Claviceps purpurea*. In transgenic *C. gracilis*, RA content increased at low temperatures, reaching 2.2 pg/cell when cultured for 7 d at 15 °C, without affecting cell growth, and was enhanced (3.3 pg/cell) by the co-expression of a palmitic acid-specific elongase gene. Most of the accumulated RA was linked with monoestolide triacylglycerol (ME TAG), in which one RA molecule was esterified to the α position of the glycerol backbone and was further esterified at its hydroxy group with a fatty acid or second RA moiety, or 1-OH TAG, in which RA was esterified to the glycerol backbone. Overall, 80% of RA was accumulated as ME TAGs. Furthermore, exogenous RA-methyl ester suppressed the growth of wild-type diatoms in a dose-dependent manner and was rapidly converted to ME TAG. These results suggest that *C. gracilis* masks the hydroxyl group and accumulates RA as the less-toxic ME TAG.

Ricinoleic acid (RA) is an unsaturated fatty acid with a double bond and hydroxyl group at positions of C9 and C12 from the carboxy end, respectively. RA is synthesised from oleic acid by fatty acid hydroxylase (FAH), which hydroxylates the carbon at position 12 in the oleic acid molecule. FAH genes have been isolated from castor bean *Ricinus communis*^1^ (*RcFAH*) as well as the fungus *Claviceps purpurea*^2^ (*CpFAH*). The RcFAH protein is localised in the endoplasmic reticulum of *R. communis*, where it preferentially hydroxylates oleic acid moieties linked at *sn*-2 positions in phosphatidylcholine^3^,^4^. Furthermore, the cellular localisation and substrate specificity of CpFAH are predicted to be similar to those of RcFAH^5^; however, CpFAH exhibits a higher sequence similarity to *C. purpurea* Δ^12^-desaturase than to RcFAH^2^.

RA serves as a precursor for the production of many bioproducts, including polyesters, biodiesel, and lubricants^6^, and castor bean seed oil is currently the only commercial source. However, castor bean is not considered agronomically suitable, because its seeds contain the harmful protein ricin, as well as allergenic 2S albumins^7^. In addition, because the ergot fungus *C. purpurea* that also produces RA is a pathogenic fungus of crop plants and grows slowly, it has been deemed unsuitable for commercial RA production. Therefore, attempts have been made to produce RA heterologously by expressing the hydroxylase gene in other oil-producing organisms. To date, heterologous RA production has been achieved by introducing the *RcFAH* gene into tobacco^1^, *Arabidopsis thaliana*^8^,^9^,^10^, and *Camelina sativa*^11^, as well as by introducing the *CpFAH* gene into *A. thaliana*^2^. To express *FAH* genes in the oilseed of *A. thaliana*^2^ and *C. sativa*^11^, seed-specific promoters were used, and when *CpFAH* expression was driven by a seed-specific promoter in the *fad2*/*fae1* mutant of *A. thaliana*, which contains elevated levels of oleic acid (a preferred substrate of FAH), the RA level accounted for up to 18% of the total seed oil content^2^.

Attempts have also been made to produce RA in microorganisms. For example, *RcFAH* has been expressed in baker’s yeast, *Saccharomyces cerevisiae*^9^, and in later studies, *CpFAH* was introduced into the fission yeast *Schizosaccharomyces pombe*^12^, *S. cerevisiae*^2^,^13^, the methylotrophic yeast *Pichia pastoris*^13^, and the oleaginous yeast *Yarrowia lipolytica*^14^. In addition, *CpFAH*-expressing *Y. lipolytica* lines have been shown to accumulate higher levels of RA than *RcFAH*-expressing lines^14^; however, the expression of CpFAH and the resulting RA production was also shown to markedly suppress cell growth in fission yeast^12^. This cellular toxicity was probably caused by the incorporation of RA into phospholipid fractions, which could affect membrane properties^12^.

At low culture temperatures, Holic *et al*.^12^ improved RA production and prevented its cellular toxicity, and Yazawa *et al*.^5^ reported that the co-expression of *CpFAH* and a phospholipase gene suppressed RA toxicity as well. Furthermore, these authors also demonstrated that phospholipase-expressing fission yeast lines secreted RA into the culture medium^15^,^16^. However, the microorganisms utilised in these studies were heterotrophs, which require exogenously added organic carbon sources to produce RA. To achieve carbon-neutral RA production based on photosynthesis without the supply of organic carbon, microalgae could be a good biological material.

In the present study, we used the oleaginous diatom *Chaetoceros gracilis*^17^,^18^ as a platform for RA production, because *C. gracilis* is used commercially as food for larval and post-larval shrimp^19^ and a transformation system for this species was established recently^20^. Here, we report that a *CpFAH*-expressing transgenic *C. gracilis* produced RA in photoautotrophic conditions, without any negative effects on cell growth, and that increased RA levels were achieved by co-expressing a palmitic acid (16:0)-specific fatty acid elongase, *Mortierella alpina* long chain fatty acid elongase1 (MALCE1). Notably, most of the synthesised RA accumulated as monoestolide triacylglycerols (ME TAGs), in which the RA hydroxyl group was masked by other fatty acids, which might explain its reduced cellular toxicity.

## Results

### Isolation of transgenic *CpFAH*-expressing *C. gracilis* cell lines

In order to produce RA in transgenic *C. gracilis* cells *via FAH* expression, a *CpFAH* cDNA fragment was obtained from cDNA pools of *C. purpurea* NBRC 6263. In an open reading frame (ORF) of *CpFAH* cloned from the NBRC 6263 strain, 13 nucleotides were found to differ from a previously reported *CpFAH* sequence (NCBI/EMBL/DDBJ accession number; EU661785^2^; Supplementary Fig. S1), and one of these polymorphisms caused an amino acid substitution A327T (Supplementary Fig. S2). Therefore, the enzymatic activity of the encoded protein was determined by heterologous expression in *S. cerevisiae* cells. The transgenic *S. cerevisiae* cell line harbouring *CpFAH* accumulated significant amounts of RA (Supplementary Fig. S3). Two Δ^12^-desaturated fatty acids: 9,12-hexadecadienoic acid (16:2Δ^9,12^) and linoleic acid (LA, 18:2Δ^9,12^) were also detected in the *CpFAH*-expressing line (Supplementary Fig. S3). These three fatty acids did not present in the vector control line.

**Figure 1:**
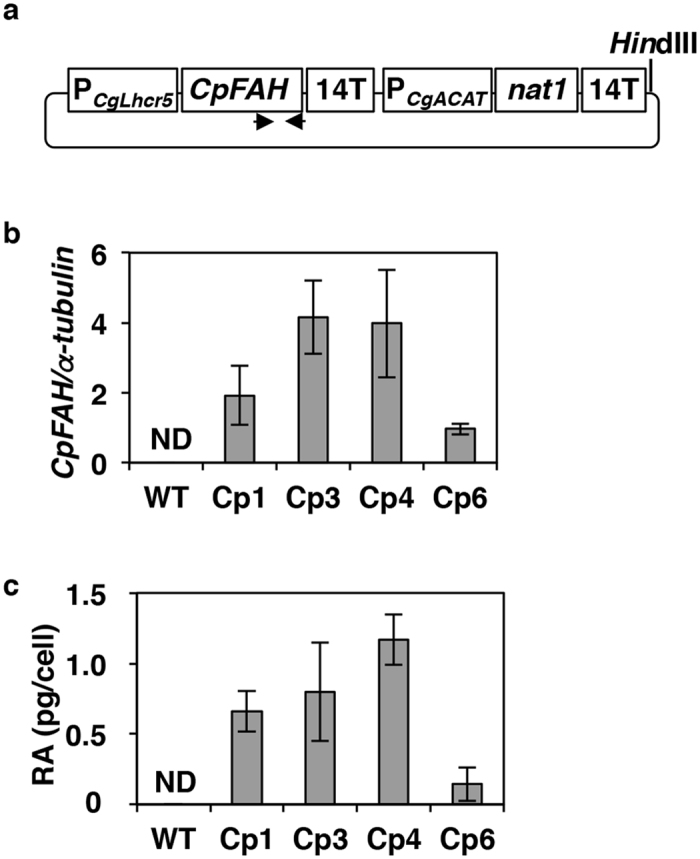
Ricinoleic acid production in transgenic lines expressing the *CpFAH* gene. (**a**) Structure of *CpFAH*-expression plasmid. A *Hin*dIII site for linearisation of the plasmid DNA before transformation into *Chaetoceros gracilis* cells is shown. Annealing sites of *CpFAH*-specific primers using genomic PCR are shown by arrows. (**b**) Expression level of *CpFAH* normalised by expression of the endogenous α*-tubulin* gene in wild-type (WT) cells and the four transgenic lines (Cp1, Cp3, Cp4, and Cp6) on d 3 °C at 20 °C. (**c**) Amount of ricinoleic acid (RA) in WT and transgenic cells after culturing for d 7 at 20 °C. Data in all experiments indicate mean value ± SD from three biological replicates. 14T, terminator of *C. gracilis Lchr14* gene; *nat1*, clonNAT resistant gene, *nourseothricin acetyltransferase* in *Streptomyces noursei*; ND, not detected; P_*CgLhcr5*_, promoter of *C. gracilis Lhcr5* gene; P_*CgACAT*_, promoter of *C. gracilis acetyl-CoA acyltransferase* gene.

After confirming the enzymatic activity of CpFAH, the *CpFAH* ORF was cloned into an expression plasmid under the control of the promoter of the fucoxanthin chlorophyll a/c-binding protein 5 (*Lhcr5*) gene from *C. gracilis* with a clonNAT-resistant gene expression cassette, which was used as a selection marker. The resulting expression plasmid (pLhcr5p-CpFAH; Fig. 1a) was used to transform *C. gracilis* cells by electroporation^20^, and four independent transgenic lines (Cp1, Cp3, Cp4, and Cp6) that contained the *CpFAH*-expression cassette were selected from the 11 clonNAT-resistant transformants using genomic PCR (Supplementary Fig. S4).

Both quantitative reverse transcription PCR (qRT-PCR) and gas chromatography–mass spectrometry (GC-MS) analyses confirmed that all four transgenic lines expressed *CpFAH* (Fig. 1b) and produced RA (Fig. 1c, Supplementary Fig. S5). In addition, the hydroxyl fatty acid (12OH-16:1Δ^9^), which was synthesised from 16:1Δ^9^ via CpFAH-catalysed hydroxylation, was also detected at 8.6 min (Supplementary Fig. S5), and the MS profiles of their trimethylsilyl derivatives including three diagnostic fragments at *m*/*z* 159, *m*/*z* 270, and *m*/*z* 299 were identical to those reported previously^2^ (Supplementary Fig. S5). Of the four lines, Cp4 exhibited the highest expression of *CpFAH* after 3 d, and the largest accumulation of RA (1.2 pg/cell) after 7 d in aerated culture at 20 °C (Fig. 1b,c). Thus, line Cp4 was used for further analyses.

### Low-temperature-dependent ricinoleic acid production in Cp4

**Figure 2:**
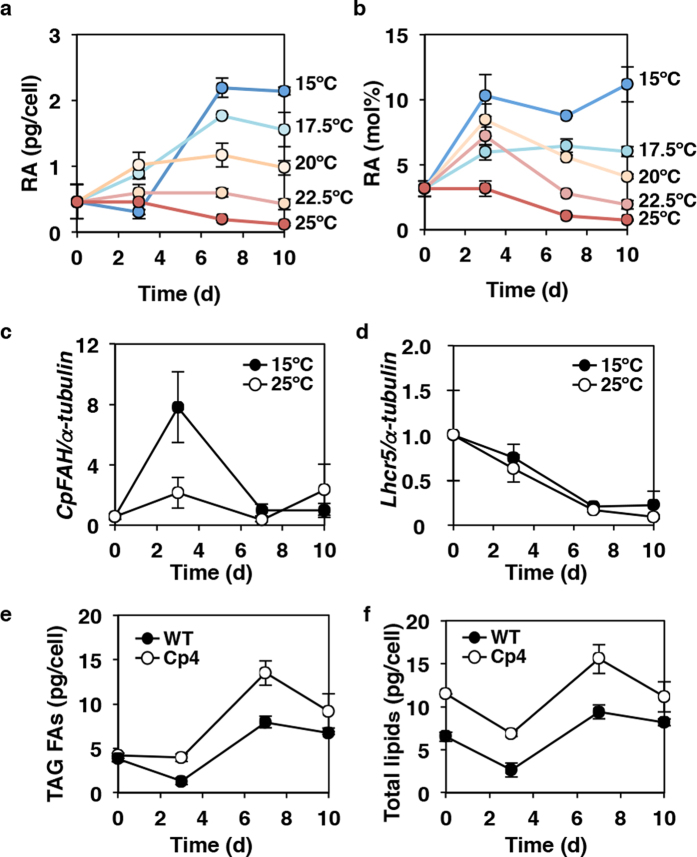
Temperature-dependent accumulation of ricinoleic acid in Cp4 cells. (**a**,**b**) Time-dependent changes in ricinoleic acid (RA) content per cell (**a**) or as percent of total lipids (**b**) in Cp4 line cultured at 15 °C, 17.5 °C, 20 °C, 22.5 °C, and 25 °C. *CpFAH* (**c**) and *Lhcr5* (**d**) gene expression in Cp4 cells cultured at 15 °C and 25 °C. Amounts of fatty acids (FAs) derived from triacylglycerol (TAG) (**e**) and total lipids (**f** ) in wild-type (WT) and Cp4 cells at 15 °C.

Optimal temperature conditions for RA accumulation were determined using Cp4 cells cultured at seven different temperatures: 10.0 °C, 12.5 °C, 15.0 °C, 17.5 °C, 20.0 °C, 22.5 °C, and 25 °C. At 10.0 °C and 12.5 °C, the cells grew poorly, and the cell density (absorbance at 730 nm) failed to reach 1.0, even after 10 d (Supplementary Fig. S6). Meanwhile, at 15 °C, Cp4 cells grew slowly but their cell density reached the same levels as observed at optimal temperatures (20–25 °C)^21^ after 7 d (Supplementary Fig. S6), In addition, we observed that the cellular content of RA and the proportion of RA in total lipids (TLs) increased in a low-temperature-dependent manner (Fig. 2a,b), with the highest cellular RA content (2.2 pg/cell; 8.8% of TLs) observed after 7 d at 15 °C (Fig. 2a, Supplementary Fig. S7).

Furthermore, the growth rates of Cp4 and wild-type (WT) cells were not significantly different at any of the temperatures examined (Supplementary Fig. S6), and the level of *CpFAH* expression in Cp4 cells increased transiently during the first 3 d at 15 °C to levels higher than those observed in cells grown at 25 °C (Fig. 2c). Conversely, the mRNA levels of endogenous *Lhcr5* expression decreased gradually at both 15 °C and 25 °C (Fig. 2d), and the levels of fatty acids derived from TAG and fatty acids derived from TLs in Cp4 cells were greater than those in WT cells after 7 d at 15 °C (Fig. 2e,f).

### Characterisation of estolide triacylglycerol structures containing ricinoleic acid

**Figure 3:**
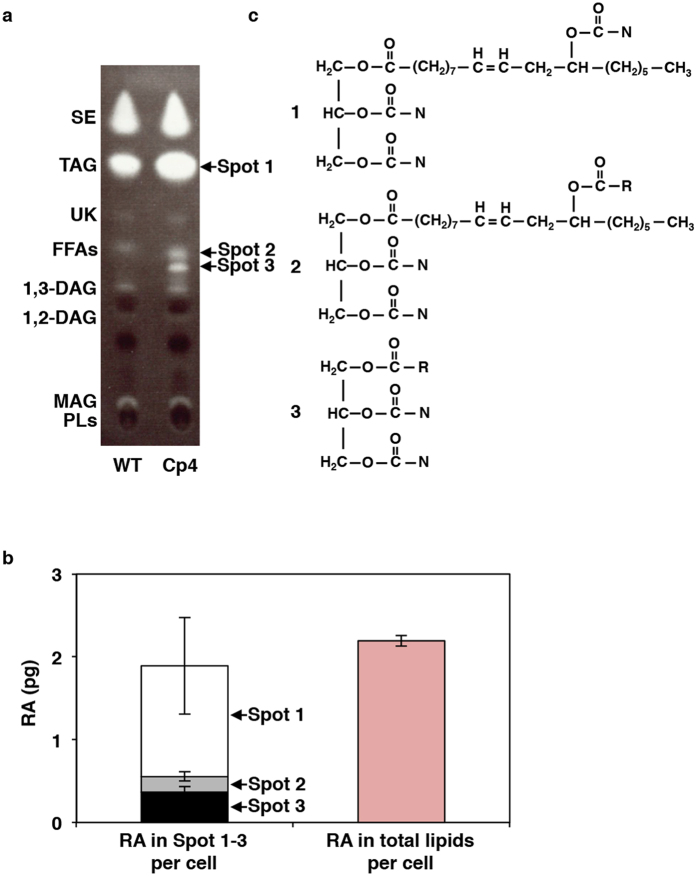
Three types of triacylglycerol containing ricinoleic acid were accumulated in Cp4 cells growing at 15 °C. (**a**) This-layer chromatography (TLC) analysis of lipid extracts from wild-type (WT) and Cp4 lines. Three Cp4-specific spots (No. 1–3), in which ricinoleic acid (RA) was detected, are indicted by arrows. Spot positions of lipid standards (1,2-DAG, 1,2-diacylglycerol; 1,3-DAG, 1,3-diacylglycerol; FFAs, free fatty acids; MAG, monoacylglycerol; PLs, polar lipids; SE, sterol ester; TAG, triacylglycerol; UK, unknown) are shown on the left side. (**b**) Distribution pattern of RA in each spot (left) and total content of RA (right) in Cp4 cells on d 7. (**c**) Structures of three types of TAG molecules containing RA extracted from each spot in (**a**). 1. mono-estolide (ME) TAG, 2. 1-OH ME TAG, and 3. 1-OH TAG. These structures were identified by liquid chromatography combined with tandem mass spectrometry (LC-MS/MS) analysis shown in Fig. 4 and Supplemental Figs S9–S10. N, acyl chain from endogenous normal fatty acids (mainly 14:0, 16:0, and 16:1); R, acyl chain from ricinoleic acid, WT, wild-type.

To identify the lipid compounds containing RA, TLs were extracted from WT and Cp4 cells cultured for 7 d at 15 °C and separated by thin-layer chromatography (TLC). Each of the resulting lipid spots, a spot of origin, and the other portions without spots was then extracted and analysed by GC-MS (Supplementary Fig. S8). Consequently, RA was detected only in the extracts of three spots (Nos 1, 2, and 3 in Fig. 3a), which contained 61% (1.3 pg/cell), 9% (0.2 pg/cell), and 17% (0.4 pg/cell) of the total cellular RA (2.2 pg/cell), respectively (Fig. 3b). In contrast, RA was not detected in the extracts from the origin, the other lipid spots, or any other parts of the TLC. In addition, the Rf values of spots Nos 1 and 2 were identical to those of normal TAG, in which three endogenous fatty acids were linked with a glycerol backbone, and free fatty acids (FFAs), respectively, and the signal intensities of the TAG and FFA spots from Cp4 cells were notably higher than those from WT cells, whereas spot No. 3 was only detected in Cp4 cells (Fig. 3a).

**Figure 4:**
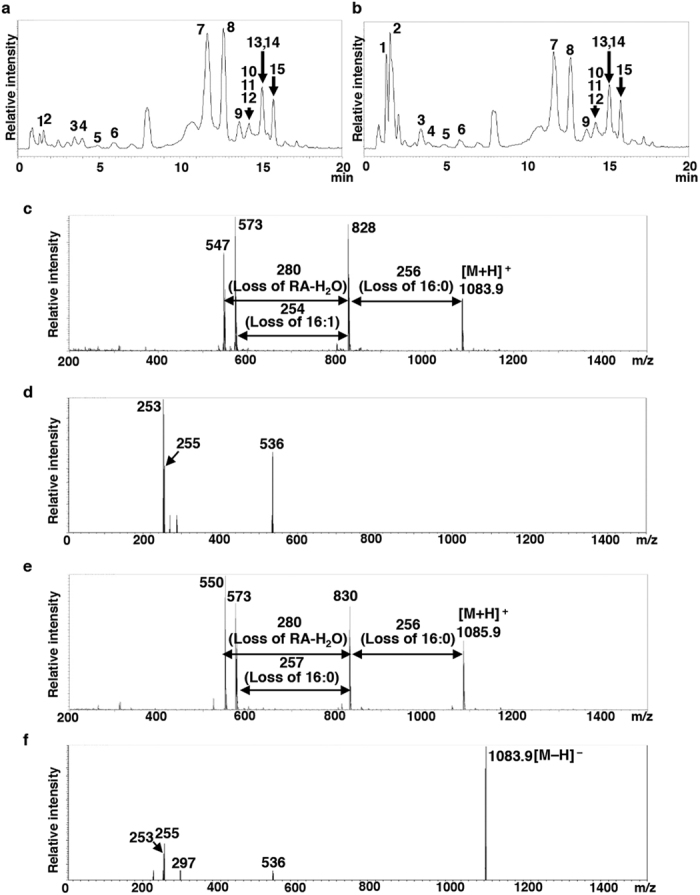
**Liquid chromatography coupled with tandem mass spectrometry analysis of spot No. 1 containing ricinoleic acid in Cp4 cells (from in** Fig. 3). Total ion chromatography in atmospheric pressure chemical Ionisation positive (**a**) and negative (**b**) modes, respectively. Each peak annotation was shown in Table 1. Six ricinoleic acid (RA)-containing triacylglycerol (TAG) species were identified as mono-estolide triacylglycerol (ME TAG; peaks Nos 10–15). The liquid chromatography combined with tandem mass spectrometry (LC-MS/MS) profiles of two major peaks, Nos 14 and 15 are shown in (**c**) to (**e**). (**c**) Full scan profile of peak No. 14 at 15.1 min in electrospray ionisation (ESI) positive mode by AutoMSMS measurement. The fragment masses and ratios are consistent with RA-16:0 estolide and two 16:1 as each side-chain fatty acid in TAG. (**d**) Scan profile of peak No. 14 with +15 V fragmentor voltage in ESI negative mode. Three fragment ions were detected at m/z = 253, 255 and 536 corresponding to 16:1, 16:0 and dehydrated RA-16:0 estolide, respectively. (**e**) Full scan profile of a peak No. 15 at 15.8 min in ESI positive mode. The fragment masses and ratios are consistent with a RA-16:0 estolide and 16:0 and 16:1 as each side-chain fatty acid in TAG. (**f** ) Scan profile of the peak No. 15 with +15 V fragmentor voltage in ESI negative mode. Three fragment ions were detected at m/z = 253, 255, 297, and 536 corresponding to 16:1, 16:0, RA, and dehydrated RA-16:0 estolide, respectively.

The structures of the RA-containing lipid molecules from each spot were determined using liquid chromatography coupled with tandem mass spectrometry (LC-MS/MS) (Table 1, Fig. 4, Supplementary Figs S9 and S10). Consequently, the RA-containing lipids from spots designated as Nos 1 and 2 in Fig. 3a were identified as ME TAG, which was the reported previously from ergot oil^22^. In these molecules, one RA molecule was linked to the glyceride backbone of TAG, and an additional fatty acids were esterified with the hydroxy group of the intracellular RA moiety (Fig. 3c).

In the LC-MS/MS analysis, total six species of ME TAG were identified from the spot No. 1 extract (Table 1). Among these, an endogenous 16:0 or 16:1 fatty acid was esterified with the hydroxy group of the RA moiety (Table 1), whereas in ME TAG detected from spot No. 2, the second RA was esterified with the hydroxyl group of the RA moiety (Fig. 3c, Supplementary Fig. S9). Therefore, we denoted ME TAG from spot No. 2 as 1-OH ME TAG. In addition, we also observed that ME TAG and 1-OH ME TAG co-migrated with TAG and FFAs, respectively. The RA-containing lipid molecules from spot No. 3 were identified as 1-OH TAG, in which an RA moiety was linked with the glyceride backbone of TAG (Fig. 3c, Supplementary Fig. S10).

To determine the RA-linked position on the ME TAG glycerol backbone, a lipid extract from spot No. 1 was hydrolysed using a site-specific lipase from *Rhizopus arrhizus* (Supplementary Fig. S11), which hydrolyses acyl chains at the α (*sn*-1 and *sn*-3) positions of lipids. Following lipase treatment, we observed that the signal intensity of the TAG spot decreased; however, the intensities of the monoacylglycerol (MAG) and FFAs increased (lane 3 in Supplementary Fig. S11). In addition, RA and 12OH-16:1Δ^9^ were detected from the FFAs spot but not from the MAG spot in the lipase-treated sample (Supplementary Fig. S11).

### Effect of exogenous hydroxyl fatty acids on cell growth of *C. gracilis*

**Figure 5:**
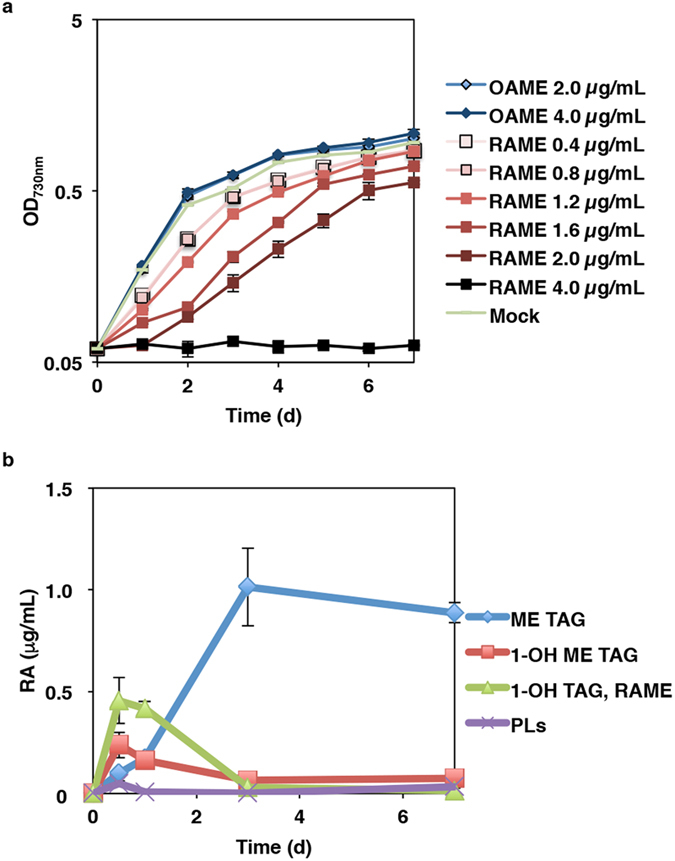
Dose-dependent effect of supplied exogenous ricinoleic acid on growth of diatom cells. (**a**) Growth curve of the wild-type line supplemented with ricinoleic acid (RA) methyl ester (RAME) or oleic acid methyl ester (OAME) in the culture medium, respectively. (**b**) Change in RA amount integrated into glycerolipids in WT cells supplemented with 1.2 μg/ml RAME. Mock, addition of solvent (ethanol) as control. MAG, monoacylglycerol; PLs, polar lipids; ME TAG, monoestolide triacylglycerol.

Previously, RA production was shown to severely suppress the cell growth of *CpFAH*-expressing fission yeast^12^. However, in the present study, the growth of Cp4 and WT *C. gracilis* cells were similar (Supplementary Fig. S6). In order to evaluate the toxicity of RA in *C. gracilis* cells, various concentrations of exogenous RA-methyl ester (RAME) was added to WT cultures at 15 °C, and oleic acid-methyl ester (OAME), which has the same structure as RAME, except for the lack of the hydroxy group, was used as a control. In addition, we used the methyl ester of each fatty acid, because the carboxy groups in FFAs are generally toxic to cells^23^,^24^, and acyl-CoA esters are unstable in the neutral aqueous solution, that is, the diatom’s medium conditions^25^. Consequently, RAME inhibited cell growth in a dose-dependent manner (Fig. 5a), and addition of 4.0 μg/ml RAME arrested cell growth completely, whereas OAME had no effect on cell growth (Fig. 5a).

To find out the destination of exogenously fed RAME in different lipid classes inside the WT cell, cellular lipid extracts from WT cells that had been cultured with 1.2 μg/ml RAME were separated using TLC, and the content of RA in each spot was quantified (Fig. 5b). RA was not detected from cells harvested immediately after the addition of RAME to the medium. At d 0.5, RA was detected in four lipid spots, which corresponded to the expected migration of ME TAG, 1-OH ME TAG, 1-OH TAG co-migrating with RAME, and polar lipids. In particular, 38% and 20% of the exogenous RA was incorporated into 1-OH TAG (0.46 μg/ml culture) and 1-OH ME TAG (0.24 μg/ml culture), respectively. Their content decreased time-dependently and became undetectable at d 3 and d 7 (Fig. 5b). Furthermore, only the level of ME TAG increased during the first 3 d, whereas the other RA-containing lipids decreased in a time-dependent manner (Fig. 5b). Consequently, 83% of the exogenously fed RAME had been incorporated into the ME TAG (1.01 μg/ml culture) at d 3, and this RA level in the ME TAG was still maintained at d 7 (0.89 μg/ml culture). At d 7, the cell density of the RAME-treated culture reached 0.9, which was the same as that observed in the control and OAME-treated cultures.

### Co-expression of *CpFAH* and *MALCE1* encoding C16-fatty acid-specific elongase

Oleic acid (18:1Δ^9^) only made a minor contribution to the fatty acid composition of transgenic *C. gracilis* cells (2.6% of total fatty acids in Cp4; Supplementary Fig. S7) but, nonetheless, it might limit the cellular content of RA. Similarly, stearic acid (18:0) was also identified as a minor fatty acid (4.2%) in Cp4 cells; whereas 16:0 was the most abundant (52.4%; Supplementary Fig. S4). Because oleic acid is synthesised from 16:0 by sequential fatty acid elongation and Δ^9^-desaturation, to increase the level of intra-cellularly accumulated RA, we attempted to increase the levels of C18 fatty acids, by providing additional 18:0 and 18:1Δ^9^ as fatty acid substrates for CpFAH-catalysed hydroxylation.

**Figure 6:**
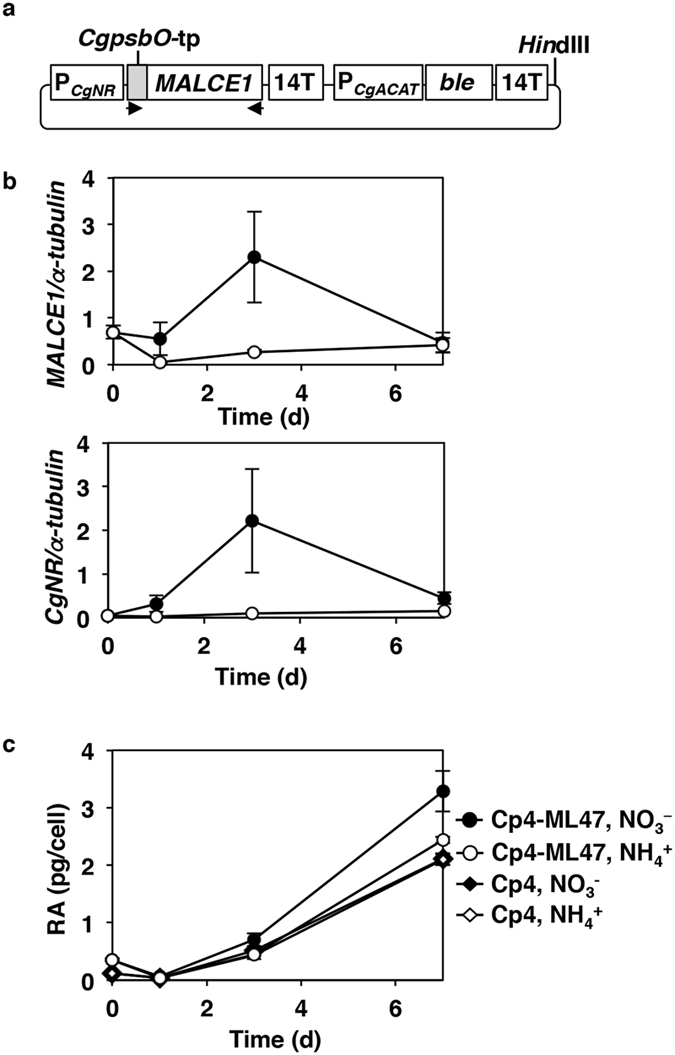
Expression and Lipid analyses of *CpFAH*- and *MALCE1*-co-expression lines. (**a**) Structure of *MALCE1*-expression plasmid. *MALCE1* gene was fused with a chloroplast transit signal from the *CgpsbO* gene and cloned under the control of the NO_3_^−^-inductive *CgNR* promoter. A *Hin*dIII site for linearisation and primer sites (arrow heads) using genomic PCR are shown. (**b**) Expression level of exogenic *MALCE1* and endogenic *CgNR* normalised by expression of the endogenous α-tubulin gene in a transgenic line, Cp4-ML47 isolated from the second transformation of the *MALCE1*-expression plasmid to Cp4 line. The cells were cultured in normal Daigo’s IMK medium containing NO_3_^−^ (filled circles) or modified Daigo’s IMK medium containing NH_4_^+^ (open circles). (**c**) Change in ricinoleic acid (RA) amount in the Cp4-ML47 and parental Cp4 lines. P_*CgNR*_, promoter of *Chaetoceros gracilis* nitrate reductase (*CgNR*) gene; P_*CgACAT*_, promoter of *C. gracilis* acetyl-CoA acyltransferase gene; *CgpsbO*-tp, chloroplast transit signal of *CgpsbO* gene; *Sh ble*, Zeocin-resistance gene.

To achieve this goal, a fungal gene encoding C16-FA-specific elongase, *MALCE1*^24^, was introduced into the transgenic Cp4 cells using an additional expression plasmid construct with a Zeocin-resistance gene, *Streptoalloteichus hindustanus* bleomycin (*Sh ble*), as a second selection marker, and the promoter region of an endogenous nitrate reductase gene^20^ (*CgNR* promoter; Fig. 6a). In addition, a partial DNA fragment of *CgpsbO* (132 bp), which encodes a chloroplast transit peptide, was fused to *MALCE1* (*CgpsbO-tp-MALCE1*; Fig. 6a) for expression of the MALCE1 protein in the chloroplast, in which 16:0 is synthesised by *de novo* biosynthesis. From screening 62 Zeocin-resistant transformants with Cp4 backgrounds using genomic PCR, seven lines (Cp4-ML17, 18, 20, 25, 43, 45, and 47) that contained the *CgpsbO*-tp*-MALCE1* expression cassette were identified (Supplementary Fig. S12), and Cp4-ML47, which accumulated the highest level of RA after 7 d at 15 °C (Supplementary Fig. S12), was subject to further analyses.

The cellular growth rate of the transgenic line Cp4-ML47 was similar to those of WT and parental Cp4 cells, when cultured in the presence of nitrate (i.e. inductive conditions) or ammonium ions (i.e. non-inductive conditions; Supplementary Fig. S12). In addition, the expression of *MALCE1* was upregulated during the first 3 d in inductive conditions, similar to the expression pattern observed for endogenous *CgNR* (Fig. 6b), and even in non-inductive conditions the level of *MALCE1* expression was slightly higher at 7 d than at 1 d (Fig. 6b). In these conditions, the RA content of the Cp4-ML47 line (3.3 pg/cell) after 7 d at 15 °C and in inductive conditions was 1.4- and 1.5-fold greater than that of the Cp4-ML47 (2.4 pg/cell) and parental Cp4 (2.2 pg/cell) cells in non-inductive conditions, respectively (Fig. 6c, Supplemental Fig. S13). Consistently, the proportion of RA (11.5%) in TLs of the Cp4-ML47 line in inductive conditions was 1.8-fold greater than that of the Cp4-ML47 (6.3% of TLs) in non-inductive conditions (Supplemental Fig. S14). Conversely, the 16:0 content (7.5 pg/cell) and its proportion in the TLs (42.7%) was 0.6- and 0.8-fold of that observed in non-inductive conditions (2.4 pg/cell; 52.6%) (Supplementary Figs S13 and S14). Although the proportion of 18:0 (7.8%) and 18:1Δ^9^ (2.2%) in TLs of the Cp4-ML47 line in inductive conditions was 1.3- and 3.8-fold greater than those of the Cp4-ML47, respectively (Supplemental Fig. S14). the contents of 18:0 (1.5 pg/cell) and 18:1Δ^9^ (0.4 pg/cell) in the Cp4-ML47 line in inductive conditions were not significantly different from those in non-inductive conditions (Supplementary Fig. S13).

To demonstrate the role of increased 18:1Δ^9^ on RA synthesis in the FAH-expressing line, Cp4 and WT cells were cultured at 15 °C for 7 d with 4 μg/ml OAME. TAG content and RA of the TAG in the Cp4 cells cultured with OAME were 1.9-fold greater than those in the Cp4 line cultured without OAME (Supplementary Fig. S15). However, the proportion of RA and other fatty acids in TAG in Cp4 line was not changed depending on the supply of OAME (Supplementary Fig. S15). Similarly, by supplying OAME, the TAG content in the WT cells increased 2.2-fold, but the fatty acid composition was not affected, (Supplementary Fig. S15).

## Discussion

RA is suitable for medical and industrial uses. In this study, carbon-neutral production of RA (2.2 pg/cell, 8.8% of TLs) was achieved by expression of the *CpFAH* gene in a transgenic oleaginous diatom *C. gracilis* in photoautotrophic conditions, without any apparent negative effects on cell growth. Furthermore, co-expression of 16:0-specific fatty acid elongase MALCE1 with CpFAH increased RA levels to 3.3 pg/cell (11.5% of TLs). Sixty-one percent of the synthesised RA was accumulated as ME TAGs, in which the RA hydroxyl group was masked by other fatty acids, which might explain its reduced cellular toxicity.

The cellular level of RA in *CpFAH*-expressing *C. gracilis* increased in a low-temperature-dependent manner (Fig. 2). Accordingly, maximum RA accumulation (1.7 pg/cell) was observed after 7 d at 15 °C, which was 8-fold greater than that observed when cultured at 25 °C (0.2 pg/cell), which is the optimal growth temperature for *C. gracilis* cells. In addition, expression analysis indicated that the abundance of *CpFAH* mRNA increased during the first 3 d of culture at 15 °C (optimal temperature for RA accumulation), after which it decreased. However, such temporal increases in *CpFAH* expression were not observed in cells cultured at 25 °C, and the expression pattern of the endogenous *Lhcr5* gene^20^, the promoter of which was used to drive *CpFAH* expression, was not observed to increase in cells cultured at either 15 °C or 25 °C. These results suggested that the stability of the *CpFAH* transcript increased temporally during the first 3 d of culture at 15 °C and that it might promote RA accumulation. An increase in the proportion of RA in the TLs occurred concomitantly with this increase in *CpFAH* mRNA stability during first 3 d (Fig. 2). Although the mRNA stability was not maintained after 3 d, the cellular content of RA was increased even after 3 d, and the proportion of RA in TLs (8.8–11%) was also maintained in this period, suggesting the protein stability of CpFAH and its enzymatic activity might be maintained at a high level at 15 °C even after the mRNA stability of *CpFAH* had declined.

When WT *C. gracilis* was cultivated with RAME, cell growth was inhibited in a dose-dependent manner (Fig. 5a). In contrast, *C. gracilis* cells cultivated with OAME exhibited the same growth patterns as cells cultivated with ethanol, which indicates that the RA hydroxyl group was toxic to *C. gracilis*. Similar to the present results, RAME has been shown to inhibit cell proliferation and viability in yeast via intracellular hydroxyl groups^26^. In addition, when a lower concentration of RAME was added to cultures (1.2 μg/ml), cell growth was restored at the late growth stage (3 d to 7 d). At 3 d, 83% of exogenously fed RA was incorporated into ME TAG, and the same levels of ME TAG were maintained still at 7 d without metabolic breakdown. On the other hand, at the early stage (0.5 d), 38% and 20% of exogenous RA was incorporated into 1-OH TAG and 1-OH ME TAG, respectively. Their content decreased time-dependently and became undetectable at d 3 and d 7 (Fig. 5b). These results suggest that half of the exogenous RAME was taken up at the early stage into TAG with free hydroxyl groups, and at least a large part of these lipids might be converted to ME TAG by an endogenous metabolic process. The accumulation of ME TAG might contribute to decrease the cellular toxicity of RA and restore cell growth. Feeding experiments using ^13^C-labelled RAME as a tracer will be necessary to identify the metabolic process of exogenous RAME. Estolides in the biological samples can be extracted efficiently without change of its property^27^ using a Bligh and Dyer liquid-liquid extraction method^28^, which was adopted in this study. However, in the industrial-scale extraction and purification of the diatom oil containing ME TAG, the effects of estolides structure on the yield at each process should be validated.

Estolide TAG has been detected in the oils of *C. purpurea*, from which *CpFAH* was isolated^22^, and also in the seed oils of *Physaria* spp^29^, *Cardamine impatiens* L.^30^, and *Trewia nudiflora* L.^31^. However, the pathway of estolide TAG synthesis remains unknown, and the related biosynthetic enzyme(s) have yet to be characterised. In *CpFAH*-expressing transgenic diatom *C. gracilis*, 61% of total RA was recovered from the TAG and ME TAG-co-eluting spots, whereas the remaining 9% and 17% were recovered from the 1-OH ME TAG and 1-OH TAG spots, respectively (Figs 3 and 4, Supplementary Figs S8–S10). This suggested that *C. gracilis* possesses an acyltransferase that catalyses the esterification of RA hydroxyl groups with the carboxy end of common fatty acids or other RA molecules. Furthermore, because RA moieties were only observed to occur at the α-position of the TAGs in *CpFAH*-expressing *C. gracilis*, ME TAG may be produced in one of two ways. First, esterification of RA hydroxyl groups with the carboxy end of common fatty acids may occur on phospholipid-linked RA moieties or on RA-CoA in the ER, after which the resulting estolide is integrated at the α-position of diacylglycerol (DAG), or alternatively, the RA is initially integrated into DAG to produce 1-OH TAG by diacylglycerol acyltransferase or phospholipid acyltransferase, after which common fatty acids are esterified with the hydroxyl groups of 1-OH TAG RA moieties. Because no hydroxyl fatty acids (HFAs), such as RA or estolide TAG, were detected in WT *C. gracilis* cells in the first place, it is unclear why the diatom possesses the biosynthetic activity of estolide TAG. However, we suggest that either (1) HFAs themselves, or diatom-infectious bacteria that produce HFA, are present in the habitat of *C. gracilis*, and so *C. gracilis* has a specific defence system for detoxifying exogenous HFAs; or (2) the production of estolide TAG could be a side-effect of producing acyltransferases involved in endogenous lipid metabolism. Nevertheless, the isolation and functional characterisation of the genes and enzymes involved in estolide synthesis are required to provide definitive answers to these hypotheses.

The amounts of RA and 16:0 accumulated by cells of the *MALCE1-CpFAH*-coexpression line Cp4-ML47 in inductive medium were 1.4-fold greater and 0.6-fold lower than that of the same line cultured in non-inductive medium, respectively. Consistently, the proportions of RA and 16:0 in TLs of the Cp4-ML47 line in inductive medium were 1.8-fold greater and 0.8-fold lower, respectively, than that of the same line cultured in non-inductive medium. Furthermore, the proportions of 18:0 and 18:1Δ^9^ in TLs of the Cp4-ML47 line cultured in inductive medium were significantly higher than those in non-inductive medium. These results suggested that the expression of *MALCE1* enhanced the conversion of 16:0 to 18:0 and promoted RA production. The condensing enzyme MALCE1 is normally located on ER and uses acyl-CoA as the substrate for acyl chain elongation by working with three other ER enzymes: ketoacyl-CoA reducatse, acyl-CoA dehydratase, and enoyl reductase in the fatty acid elongase complex^32^. In this study, MALCE1 was fused to a plastid-transit peptide at the N-terminus, suggesting that this enzyme can also use acyl-ACP as a substrate in cooperation with three endogenous plastidic enzymes: ketoacyl-ACP reductase, acyl-ACP dehydratase, and enoyl reductase. The accumulated levels of 18:0 and 18:1Δ^9^ in the Cp4-ML47 line did not show any difference between cells cultured in inductive and non-inductive conditions, suggesting that the Δ^9^-desaturation from 18:0 to 18:1Δ^9^ is not a rate-limiting step in the production of RA. Furthermore, the RA content of the Cp4-ML47 line in non-inductive conditions was slightly greater than that of the Cp4 line expressing the *CpFAH* gene. This might be caused by the leaky expression of *MALCE1* regulated by the *CgNR* promoter, triggered by the depletion of ammonium ions in the medium during the late growth phase.

Supply of exogenous OAME in the Cp4 culture increased the TAG and RA contents in cells (Supplemental Fig. S15), suggesting that *C. gracilis* cells could take up exogenous OAME from the medium and utilize it for endogenous lipid metabolic processes. Unexpectedly, the fatty acid composition of TAG fatty acids was not changed between the Cp4 cells cultured with OAME and without OAME. This indicated that all fatty acid moieties in TAG increased with keeping each ratio. At first, we predicted that the exogenously fed OAME enhanced only the downstream lipid metabolism, that is, production of RA by CpFAH and production of the linoleic acid and other C18- to C22-polyunsaturated fatty acids, such as EPA and DHA, by endogenous fatty acid desaturation and elongation processes. We suggest that either (1) OAME was incorporated into cellular lipids or converted to OA-CoA to synthesize downstream lipid, and endogenous supply of OA from 16:0 fatty acid was suppressed to maintain the balance of the overall fatty acid ratio; or (2) when exogenous OAME was taken up into *C. gracilis* cells, it was digested through catabolic pathways and used for *de novo* fatty acid synthesis. Actually, in WT *C. gracilis* cells cultured with the exogenous OAME, TAG contents also increased to maintain the fatty acid ratio (Supplemental Fig. S15). This suggested that *C. gracilis* maintained metabolic homeostasis of fatty acids by adapting to the supply of exogenous fatty acids.

To enhance the conversion of acyl chains, including RA, from phospholipids to TAG in *A. thaliana*, a castor phospholipid:diacylglycerol acyltransferase (PDAT) was co-expressed with the *RcFAH* gene, which resulted in an RA content of 19%^33^ in the seed oil. However, in *CpFAH*-expressing *C. gracilis*, TAG accounted for 87% of the TLs, and RA was only found in TAG molecules. Therefore, the co-expression of *PDAT* and *CpFAH* might not be necessary in *C. gracilis* considering that the TAG content of WT *C. gracilis* reaches 84% of TLs. Alternatively, use of more powerful promoters than *Lhcr5* and *NR* in this study for the expression of *CpFAH* and *MALCE1* genes, and knockout or knockdown of any endogenous Δ^12^-desaturase gene in RA-producing lines may be effective methods for further enhancing the production of RA in *C. gracilis* cells. Furthermore, TAG and total fatty acid contents in *CpFAH*-expressing *C. gracilis* cultured at 15 °C were 1.8-fold that in WT cells (Fig. 2), which has not been reported in other heterologous RA-producing organisms. Their production may have been promoted to compensate for the heterologous production of RA, as well as for the production of ME TAG molecules. These findings suggest that *CpFAH*-expressing *C. gracilis* can be used as a resource of biofuel production. Notably, *CpFAH*-expressing *C. gracilis* accumulates modest level of RA in estolide TAG without growth inhibition, potentially because estolide reduces the cellular toxicity of RA. Both estolide TAG^34^,^35^ and estolides^36^ have several other valuable chemical properties as well, which could be utilised for industrial and medical uses in the future. Therefore, we conclude that *C. gracilis* is an attractive producer of RA and estolide TAG.

## Methods

### Strains and culture conditions of *C. gracilis*

A wild-type *Chaetoceros gracilis* strain (UTEX LB2658) was used for transformation. It was cultured in Daigo’s IMK medium (Nihon Pharmaceutical, Osaka, Japan) supplemented with sea salts (Sigma-Aldrich, St. Louis, MO, USA) and 0.2 mM Na_2_SiO_3_. The cells were grown at 20 °C in continuous light conditions at 50 μmol photons/m^2^/s^1^. At the start of culture, the cell density at 730 nm was adjusted to 0.07. For feeding experiments, the stated concentration of RAME (Sigma-Aldrich) and OAME (Sigma-Aldrich) was added at the start of culture.

### cDNA isolation from *C. purpurea*

To obtain a cDNA fragment encoding the *CpFAH* gene, cDNA pool of a WT *C. purpurea* NBRC 6263 was constructed by reverse-transcription (RT). *C. purpurea* NBRC 6263 was provided by the National Institute of Technology and Evaluation (NITE). Total RNA for the RT reaction was extracted using a RNeasy mini Kit (Qiagen, Hilden, Germany) from *C. purpurea* cells cultured in C medium^37^ for 1 month at 28 °C. The resultant total RNA (0.81 μg) was subjected to an RT reaction using a PrimeScript High Fidelity RT-PCR kit (TaKaRa Bio, Shiga, Japan) in accordance with the manufacturer’s instructions. The ORF of the *CpFAH* gene was amplified for the construction of a yeast expression plasmid with gene-specific primers shown in Supplementary Table S1, and sequenced.

### Transformation of yeast cells

To express the *CpFAH* gene in yeast cells, the *CpFAH* ORF derived from *C. purpurea* NBRC 6263 was cloned under the control of a galactose-inducible *GAL1* promoter in a yeast expression vector pYES2 (Thermo Fisher Scientific, Waltham, MA, USA). *S. cerevisiae* INVSc1 (Thermo Fisher Scientific) was transformed using a lithium acetate-mediated transformation procedure^38^. The resultant clone was pre-cultured for 2 d at 28 °C in SC minimal medium and then cultured for 2 d at 20 °C in the SC minimal medium containing 2% (w/v) galactose substituted for glucose.

### Vectors construction and transformation of *C. gracilis* cells

To obtain transgenic *C. gracilis* lines expressing the *CpFAH* gene, the *CpFAH* ORF derived from *C. purpurea* NBRC 6263 was amplified by PCR using primers *CpFAH-BglII-fw* and *CpFAH-NsiI-rev* and cloned into the *Bam*HI-*Pst*I site of a pCgLhcr5p plasmid (accession number; AB981621) downstream of the *CgLhcr5* promoter for a light-harvesting fucoxanthin chlorophyll protein (*fcp*) gene^20^. The resultant plasmid was linearised by digestion using *Hin*dIII for transformation. Transformation was performed by a multi-pulse electroporation method using NEPA21 apparatus (Nepagene, Chiba, Japan)^20^,^39^. The transformed cells were selected on Daigo’s IMK agar plates containing 1% agar and 400 μg/ml nourseothricin (clonNat, Werner Bioagents, Thuringia, Germany) and further screened by genomic PCR (Supplementary Fig. S1).

To introduce MALCE1 into chloroplasts of the *CpFAH*-expressing line (Cp4), the pCgNRp plasmid (AB981622)^20^ was firstly modified to have a cDNA encoding the transit peptide (tp) region of *CgpsbO* (accession number; AB373993) downstream of the *CgNR* promoter from a nitrate reductase gene (*NR*): A 132 bp-fragment of *CgpsbO*-tp amplified using the following primers: *InFusion_NRp/CgpsbOtp_fw* and *PsbOsignal_AflII_rv*, was inserted into the *Bam*HI-*Xba*I site of the pCgNRp plasmid generating a new pCgNRp/*CgpsbO*-tp vector. Then, a fragment of the *MALCE1* gene (accession number; AB468587) amplified using primers *MALCEI_AflII_fw* and *MALCEI_XbaI_rv* was inserted into the *Afl*II-*Xba*I site of this vector. Furthermore, a clonNat-registrant gene (*nat1*) gene was replaced with a Zeocin-resistance gene (*Sh ble*) for the secondary transformation of the clonNat-resistant Cp4 line: a PCR-amplified fragment of the *Sh ble* gene using primers *Sh_ble_BglII_fw* and *Sh_ble_NsiI_rv* was subcloned into the *Bam*HI-*Pst*I site of pUC118-pCgACAT-nat downstream of the acetyl-CoA acetyltransferase (*CgACAT*) promoter (AB981624)^20^. The resulting vector was digested with *Sac*I and *Hin*dIII, and the fragment containing part of the *CgACAT* promoter and the *Sh ble* gene was transferred into the *Sac*I-*Hin*dIII site of pCgNRp/*CgpsbO*-tp-*MALCE1*. The resultant plasmid was linearised by digestion using *Hin*dIII for transformation. Transformed Cp4 cells resistant to both 400 μg/ml clonNat and 100 μg/ml Zeocin (Sigma-Aldrich) were selected and further screened by genomic PCR. The PCR primers used for vectors construction and genomic PCR selection are listed in Supplementary Table S1.

### Quantitative real-time PCR

Quantitative real-time PCR was performed using SYBR Premix Ex Taq GC (Takara Bio) and a LightCycler 480 Instrument (Roche, Basel, Switzerland), as described previously^40^. The Cg α*-tubulin* gene was used as an internal control. The primers used for qRT-PCR are described in Supplementary Table S1.

### Lipid analysis

TLs were extracted from the yeast and diatom cells in a chloroform–methanol–water system^28^. For transesterification of the TLs, the extracted TLs were incubated in 2.5% hydrogen chloride/methanol at 85 °C for 2.5 h, and fatty acid methyl esters (FAMEs) were extracted by 4 ml of petroleum ether with 0.4 ml of 5 M NaCl, and dried under N_2_ gas. 20 μl of 1 mM heptadecanoic acid (17:0) was added to the each lipid sample before the transesterification, and used as an internal standard. The resultant FAMEs dissolved in 30 μl of acetonitrile, were trimethylsilylated by adding 30 μl of *N*,*O*-bis(TMS)-acetamide:pyridine (1:1), and heating at 90 °C for 30 min. Then, 1 μl of total FAMEs and TMS derivatives were analyzed using a Shimadzu gas chromatography mass spectrometry, GCMS-QP2010 system (Shimadzu, Kyoto, Japan) equipped with a DB-23 column (30 m × 0.25 mm) with 0.25-μm film thickness (Agilent Technologies, CA, Santa Clara). The column temperature was maintained at 160 °C for 1 min, and then increased to 210 °C at a rate of 4 °C/min. For MS, the mass selective detector under electron impact conditions (70 eV) was scanning an effective *m/z* range of 40–500 at 1,666 amu/sec. Neutral lipids were separated into sterol esters, MAG, diacylglycerol, and triacylglycerol (TAG) by TLC using silica plates (TLC silica gel 60, 20 × 20 cm; Merck Millipore, Darmstadt, Germany) developed with *n*-hexane/diethyl ether/acetic acid (70:30:1 v/v). After drying, the plate was sprayed with 80% aqueous acetone containing 0.01% primuline. TAG spots were scraped for transesterification as above. Then, 20 μl of 1 mM 17:0 added to the sample before transesterification was used as the internal standard for quantification. The amount of TAG was quantified by gas–liquid chromatography (GC) using a Shimadzu (Kyoto, Japan) GC-1700 gas chromatograph equipped with a flame ionization detector and a split-less injection system, fitted with a capillary column (TC-70, 60 m × 0.25 mm I.D., GL Sciences, Tokyo, Japan) after derivatisation to FAMEs. The initial column temperature was maintained at 60 °C for 2.5 min, increased to 180 °C at range of 40 °C/min, maintained at 180 °C for 10 min, increased to 260 °C at range of 5 °C/min, and then maintained for 8.5 min. The injector and detector were operated at 250 °C. The fatty acid peaks were identified by comparing the retention times to known standards.

### Positional analysis of TAG

Positional analysis of TAG was performed using *R. arrhizus* lipase (Sigma-Aldrich) as reported previously^41^. Lipids were extracted from the *C. gracilis* cells and resolved by TLC as described above. TAG was extracted from silica gel using the chloroform–methanol–water system as above^28^. Approximately 10 mg was dried under a N_2_ gas stream and resuspended in 350 μl of 0.1 M PBS buffer (pH 7.4) containing 4.28 mM Triton X-100. The sample was dispersed by sonication (Sonicator UR-21P, Tomy) for 6 × 10 s (output control: 8) on ice. Then, 20 μg of *R. arrhizuz* lipase dissolved in the 100 μl of PBS buffer was added to the emulsified TAG preparation, and incubated at 22 °C for 3 h. The lipase-treated lipids were extracted from the reaction mixtures, and resolved by TLC then developed with *n*-hexane/diethyl ether/acetic acid (70:140:3 v/v) for the separation of lipids containing HFAs, as described previously^9^. FFAs and MAG spots were scraped for transesterification as above.

### Liquid chromatography coupled with tandem mass spectrometry analysis

LC-MS/MS analysis was performed using a LCMS-8040 tandem quadrupole mass spectrometer (Shimadzu, Kyoto, Japan). LabSolution LCMS software Ver. 5.65 (Shimadzu) was used to control the instrument and to process the data. The system used in the analysis consisted of a system controller CBM-20A, two pumps LC-30AD, and autosampler SIL-30AC, a column heater CTO-30AC, and a degasser DGU-20A_5_.

Lipids were extracted from the silica gel derived from the each TLC spot by the Bligh and Dyer method^28^ and dried by evaporation. Then, the extracts were resolved with 1,000 μl of 2-propanol and subjected to LC-MS/MS analysis. The liquid chromatography conditions were optimised as follows: solvent A was water, and solvent B was 2-propanol. The gradient profile was as follows: 100% B (1–25.0 min); 80% B (25.1–30 min). The flow rate was set to 0.2 ml/min, and the column temperature was 40 °C. Chromatographic separation was carried out on a Shim-pack XR-ODSII (75 × 2.0 mm, 2.2 μm, Shimadzu GLC, Tokyo, Japan). The injection volume was 5 μl. An LCMS-8040 tandem quadrupole mass spectrometer was operated in both positive and negative mode with APCI source in the range of m/z 200–1200. The operating parameters were optimised as follows: nebulizer gas flow, 4.0 l/min; drying gas flow, 5.0 l/min; desolvation line (DL) temperature, 200 °C; heat block temperature, 200 °C.

### Accession number of the *CpFAH* gene used in this study

The *CpFAH* sequence in *C. purpurea* NBRC 6263 used in this study was deposited in the NCBI/EMBL/DDBJ database as accession number LC149858.

## Additional Information

**How to cite this article**: Kajikawa, M. *et al*. Production of ricinoleic acid-containing monoestolide triacylglycerides in an oleaginous diatom, *Chaetoceros gracilis*. *Sci. Rep*. **6**, 36809; doi: 10.1038/srep36809 (2016).

**Publisher’s note:** Springer Nature remains neutral with regard to jurisdictional claims in published maps and institutional affiliations.

## Supplementary Material

Supplementary Information

## Figures and Tables

**Table 1:**
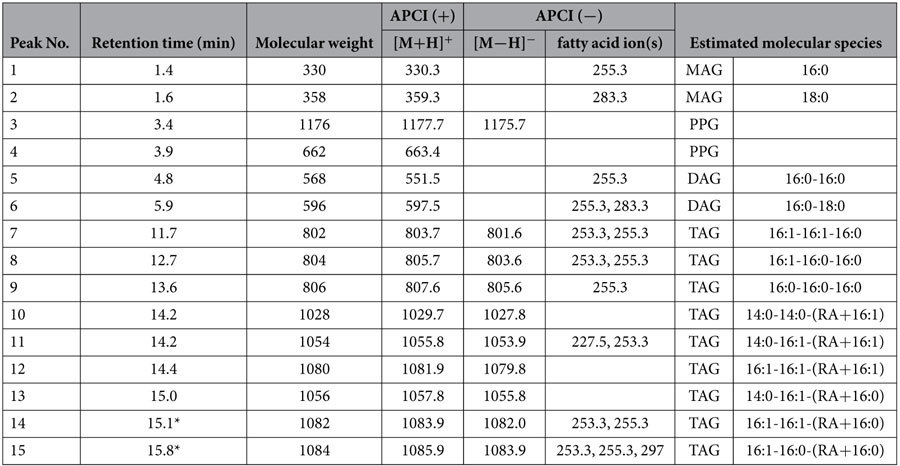
Major molecular species from Spot 1 in TLC analysis of Cp4 line shown in Fig. 3 identified by MS/MS analysis.

Individual structures of these lipid molecular species were estimated by the MS/MS analysis. *MS/MS profiiles of peaks at 15.1 and 15.8 min are shown in Fig. 4 as representatives of monoestolide (ME) TAG. APCI, atmospheric pressure chemical ionization; DAG, diacylglycerol; MAG, monoacylglycerol; PPG, polypropyleneglycol; TAG, triacylglycerol.
