# Supplementary material for: Production of ricinoleic acid-containing monoestolide triacylglycerides in an oleaginous diatom, Chaetoceros gracilis
Source: Sci Rep. 2016 Nov 10;6:36809. doi: 10.1038/srep36809 (PMC5103293; doi:10.1038/srep36809)
Supplement: Supplementary Information [file srep36809-s1.pdf]

Production of ricinoleic acid-containing monoestolide triacylglycerides in an oleaginous diatom, *Chaetoceros gracilis*

Masataka Kajikawa[1], Tatsuki Abe[1], Kentaro Ifuku[1], Ken-ichi Furutani[1], Dongyi Yan[1], Tomoyo Okuda[2], Akinori Ando[2], Shigenobu Kishino[2], Jun Ogawa[2] & Hideya Fukuzawa[1]

[1]Graduate School of Biostudies, Kyoto University, Kyoto 606-8502, Japan.
[2]Graduate School of Agriculture, Kyoto University, Kyoto 606-8502, Japan.
Correspondence and requests for materials should be addressed to H.F. (email: fukuzawa@lif.kyoto-u.ac.jp)

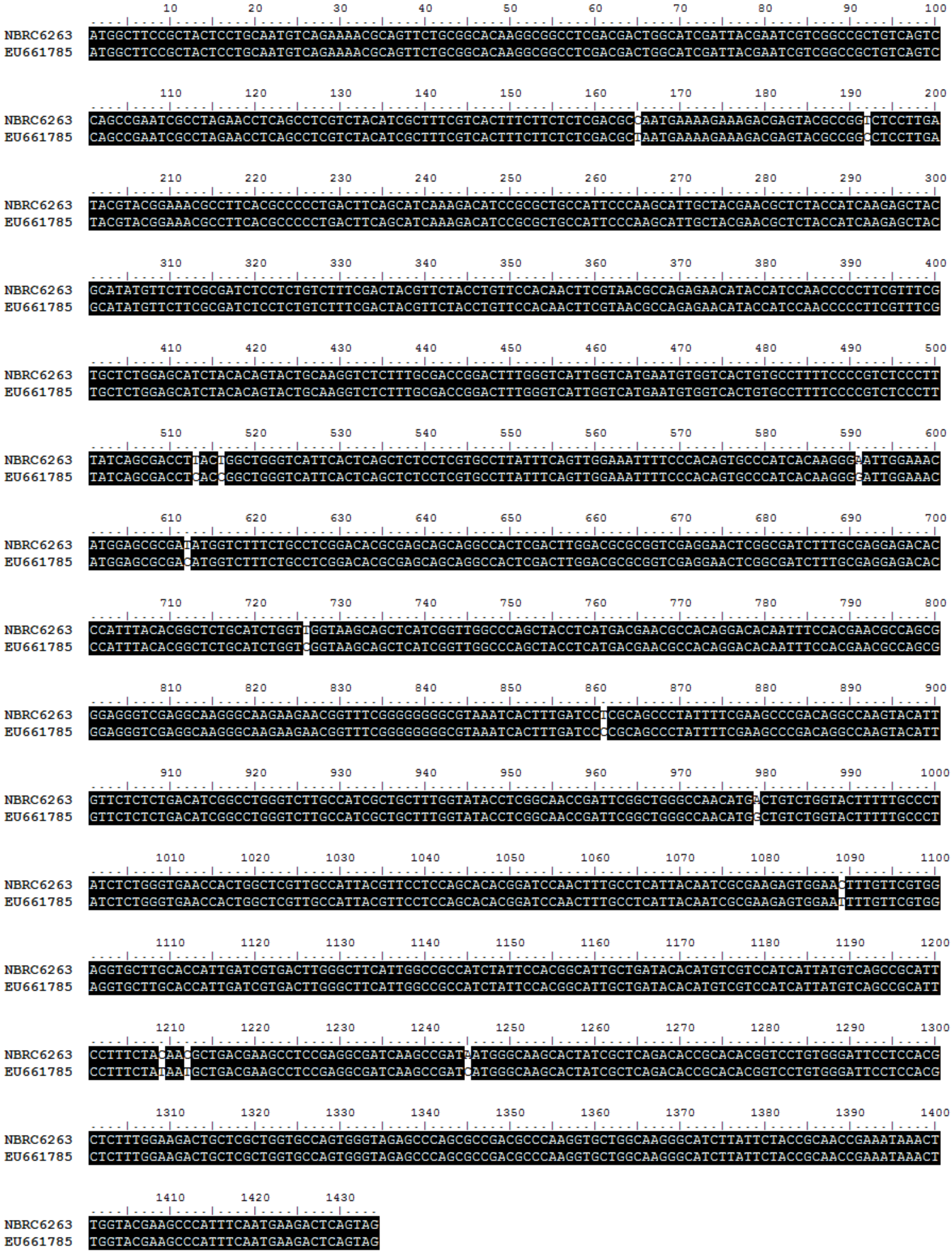

Supplementary Fig. S1: Alignment of coding sequences of a *CpFAH* gene isolated from a *Claviceps purpurea* NBRC 6263 (in this study) and the *CpFAH* gene (NCBI/EMBL/DDBJ accession number; EU661785) reported previously[2]. Identical nucleotides between the two sequences are boxed.

```
                10        20        30        40        50        60        70        80        90       100
       ....|....|....|....|....|....|....|....|....|....|....|....|....|....|....|....|....|....|....|....|
NBRC6263 MASATPAMSENAVLRHKAASTTGIDYESSAAVSPAESPRTSASSTSLSSLSSLDANEKKDEYAGLLDTYGNAFTPPDFSIKDIRAAIPKHCYERSTIKSY
EU661785 MASATPAMSENAVLRHKAASTTGIDYESSAAVSPAESPRTSASSTSLSSLSSLDANEKKDEYAGLLDTYGNAFTPPDFSIKDIRAAIPKHCYERSTIKSY

               110       120       130       140       150       160       170       180       190       200
       ....|....|....|....|....|....|....|....|....|....|....|....|....|....|....|....|....|....|....|....|
NBRC6263 AYVLRDLLCLSTTFYLFHNFVTPENIPSNPLRFVLWSIYTVLQGLFATGLWVIGHECGHCAFSPSPFISDLTGWVIHSALLVPYFSWKFSHSAHHKGIGN
EU661785 AYVLRDLLCLSTTFYLFHNFVTPENIPSNPLRFVLWSIYTVLQGLFATGLWVIGHECGHCAFSPSPFISDLTGWVIHSALLVPYFSWKFSHSAHHKGIGN

               210       220       230       240       250       260       270       280       290       300
       ....|....|....|....|....|....|....|....|....|....|....|....|....|....|....|....|....|....|....|....|
NBRC6263 MERDMVFLPRTREQQATRLGRAVEELGDLCEETPIYTALHLVGKQLIGWPSYLMTNATGHNFHERQREGRGKGKKNGFGGGVNHFDPRSPIFEARQAKYI
EU661785 MERDMVFLPRTREQQATRLGRAVEELGDLCEETPIYTALHLVGKQLIGWPSYLMTNATGHNFHERQREGRGKGKKNGFGGGVNHFDPRSPIFEARQAKYI

               310       320       330       340       350       360       370       380       390       400
       ....|....|....|....|....|....|....|....|....|....|....|....|....|....|....|....|....|....|....|....|
NBRC6263 VLSDIGLGLAIAALVYLGNRFGWANMTVWYFLPYLWVNHWLVAITFLQHTDPTLPHYNREEWNFVRGGACTIDRDLGFIGRHLFHGIADTHVVHHYVSRI
EU661785 VLSDIGLGLAIAALVYLGNRFGWANMAVWYFLPYLWVNHWLVAITFLQHTDPTLPHYNREEWNFVRGGACTIDRDLGFIGRHLFHGIADTHVVHHYVSRI

               410       420       430       440       450       460       470
       ....|....|....|....|....|....|....|....|....|....|....|....|....|....|..
NBRC6263 PFYNADEASEAIKPIMGKHYRSDTAHGPVGFLHALWKTARWCQWVEPSADAQGAGKGILFYRNRNKLGTKPISMKTQ
EU661785 PFYNADEASEAIKPIMGKHYRSDTAHGPVGFLHALWKTARWCQWVEPSADAQGAGKGILFYRNRNKLGTKPISMKTQ
```

Supplementary Fig. S2: Alignment of deduced amino acid sequences of CpFAH in *Claviceps purpurea* NBRC 6263 (in this study) and the CpFAH gene (NCBI/EMBL/DDBJ accession number; EU661785) reported previously[2]. Identical amino acid residues between the two sequences are boxed.

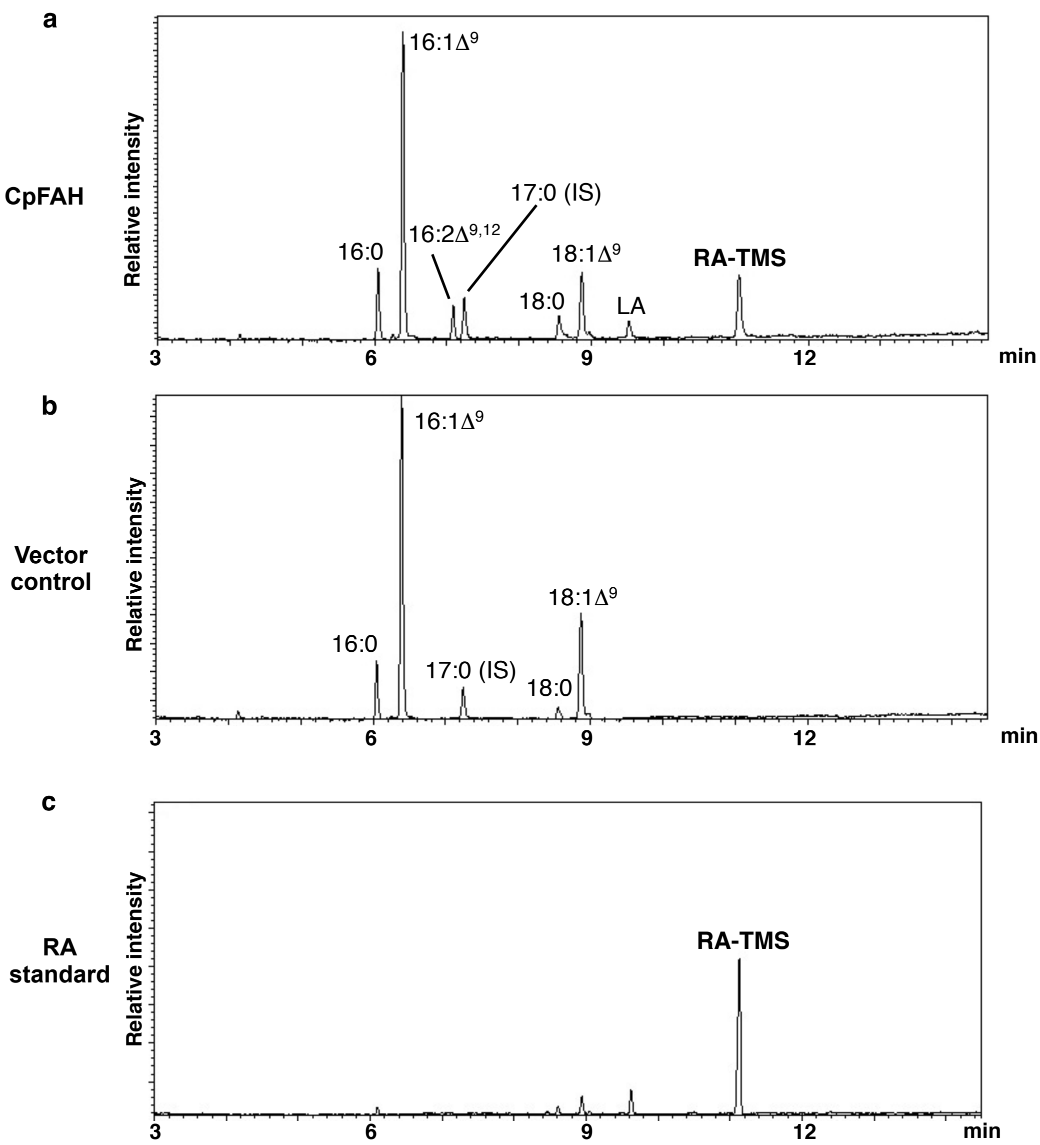

Supplementary Fig. S3: Gas chromatography (GC) analysis of fatty acid methyl ester (FAME)-trimethylsilyl (TMS) derivatives prepared from yeast expressing the *CpFAH* gene derived from wild-type *Claviceps purpurea* NBRC 6263 (**a**) or yeast transformed with an empty vector (**b**). TMS ester of ricinoleic acid (RA) was used as standard (**c**). LA, linoleic acid.

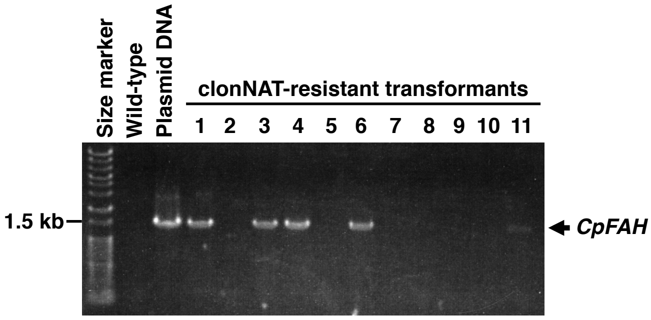

Supplementary Fig. S4: Genomic-PCR analysis of the transgenic *Chaetoceros gracilis* lines transformed with a *CpFAH*-expression plasmid. Size marker, 1-kb DNA size marker (Thermo Fischer Scientific); wild-type, genomic DNA from *C. gracilis* (negative control); Plasmid DNA, the expression plasmid DNA used for transformation (positive control).

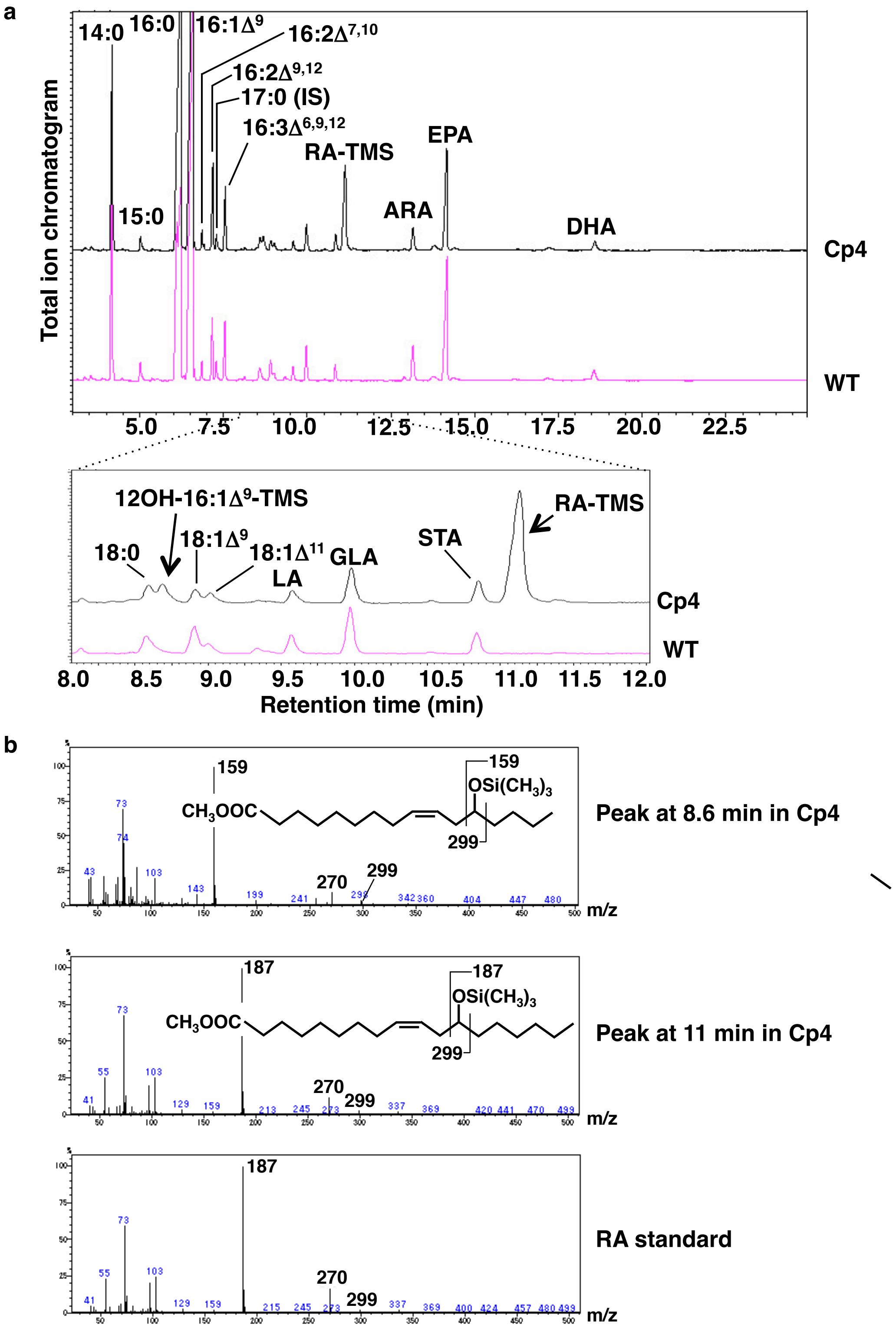

Supplementary Fig. S5: Gas chromatography–mass spectrometry (GC-MS) analysis of total lipids in wild-type (WT) and Cp4 cells culturing at 20°C for 7 d. (**a**) Total ion chromatography of fatty acid methyl ester (FAME)-trimethylsilyl (TMS) derivatives of total lipids in WT and Cp4 cells. (**b**) mass spectrometry profiles of FAME-TMS derivatives of two hydroxyl fatty acids (12OH-16:1Δ^9 and ricinoleic acid [RA]) in Cp4 cells detected at 8.6 and 11 min, respectively. A TMS ester of RA standard is shown in the bottom panel. ARA, arachidonic acid; DHA, docosahexaenoic acid; EPA, eicosapentaenoic acid; GLA, γ-linolenic acid, IS, internal standard; LA, linoleic acid; RA-TMS, trimethylsilyl ester of ricinoleic acid, STA, stearidonic acid.

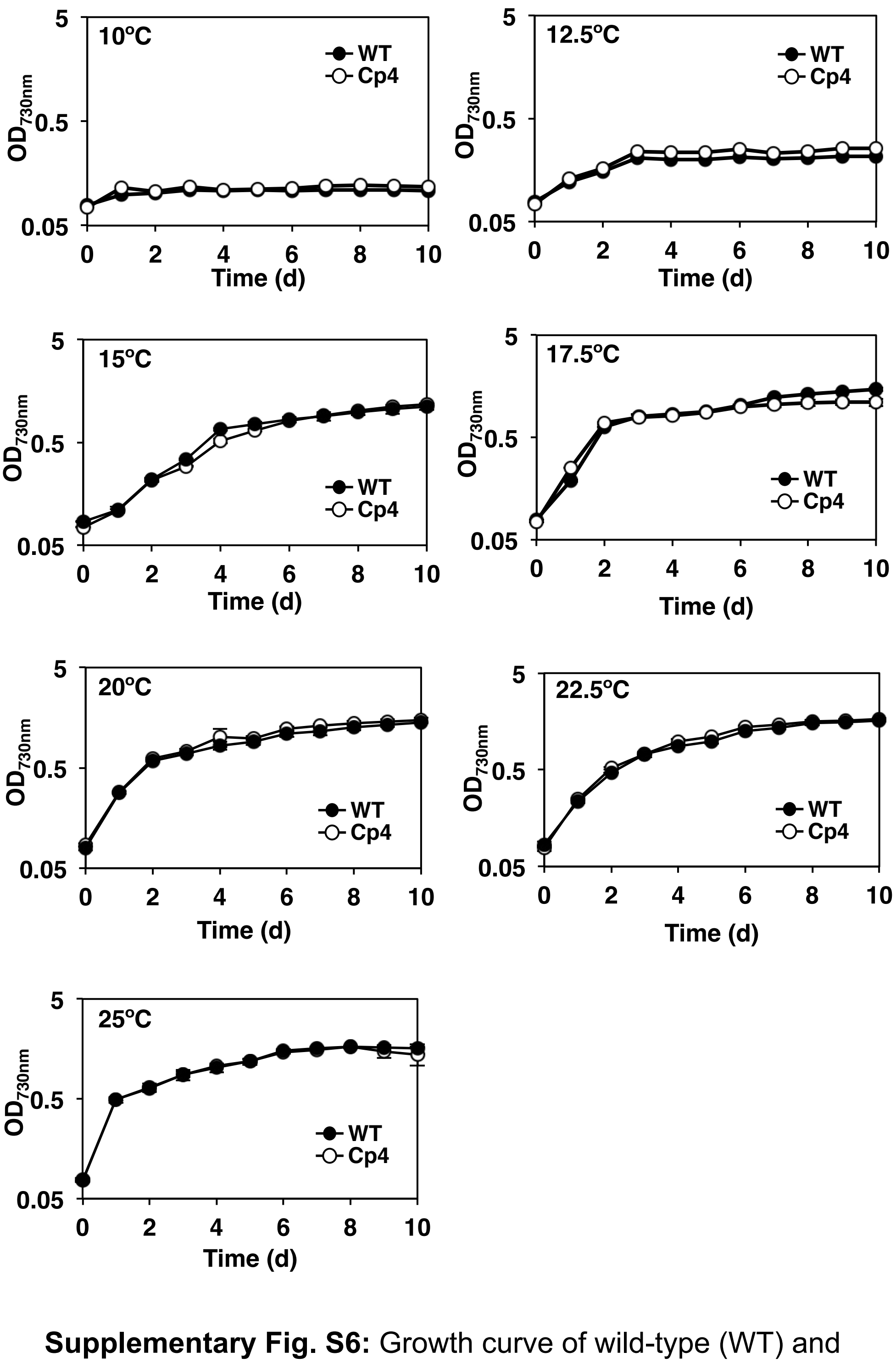

Supplementary Fig. S6: Growth curve of wild-type (WT) and Cp4 cells cultured at seven temperature conditions (10ºC, 12.5ºC, 15ºC, 17.5ºC, 20ºC, 22.5ºC, and 25ºC) for 10 d.

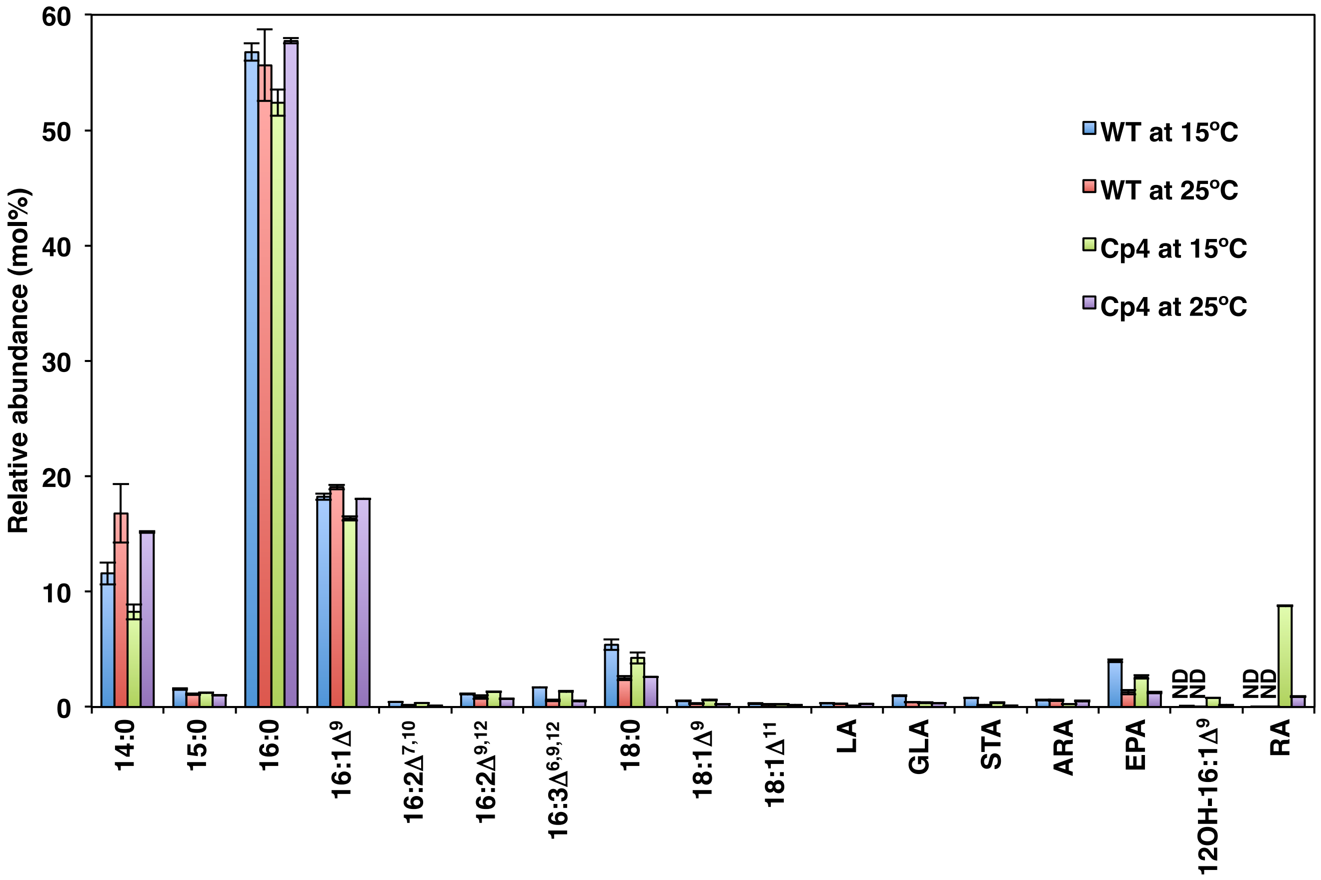

Supplementary Fig. S7: Fatty acid compositions of total lipids in wild-type (WT) and Cp4 cells cultured at 15ºC or 25ºC for 7 d. ARA, arachidonic acid; EPA, eicosapentaenoic acid; GLA, γ-linolenic acid, LA, linoleic acid; RA, ricinoleic acid; STA, stearidonic acid. ND, not detected.

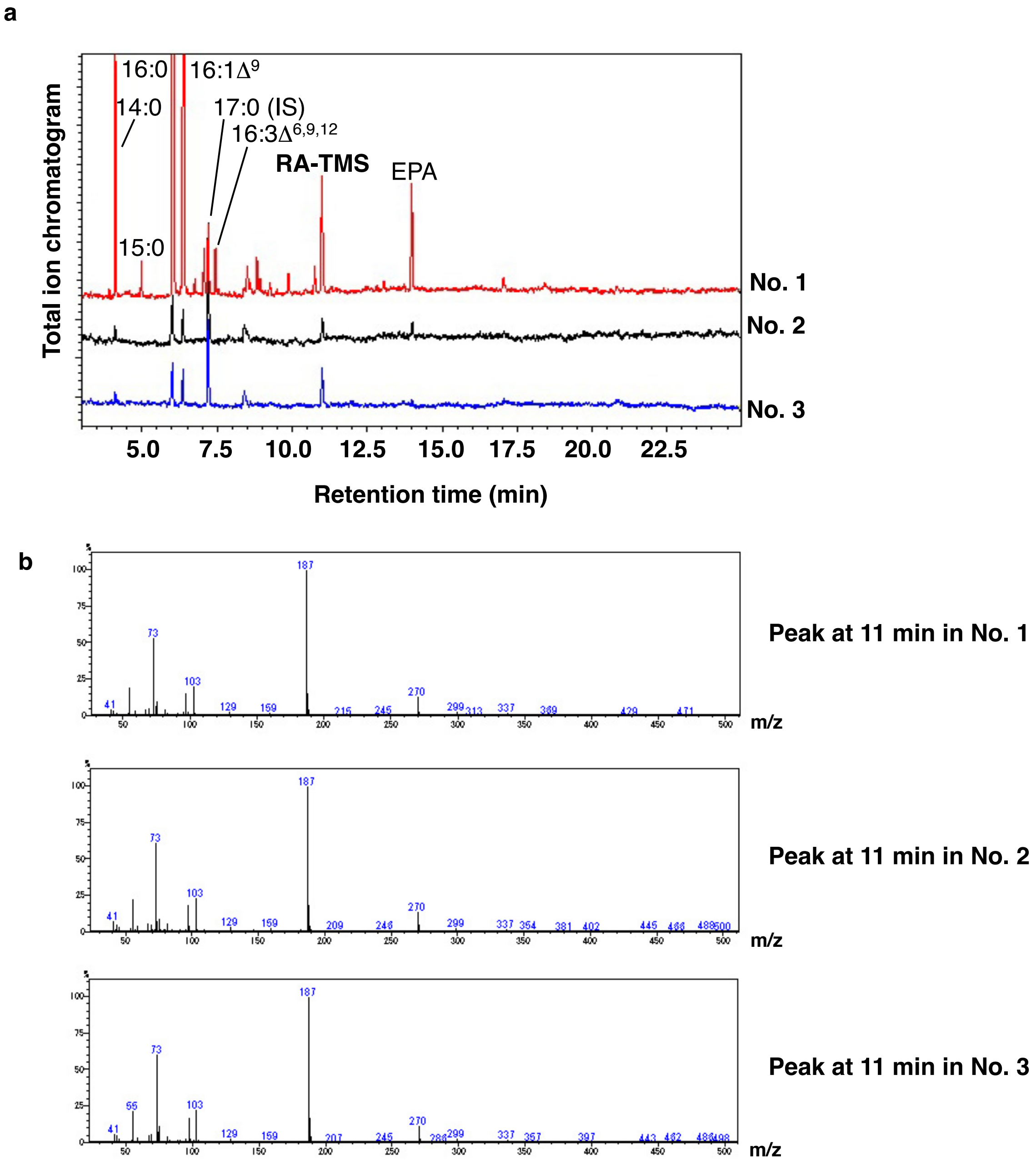

Supplementary Fig. S8: Gas chromatography–mass spectrometry (GC-MS) analysis of the lipids extracted from spots No. 1–3 in Cp4 cells shown in Fig. 3.
(**a**) Total ion chromatography of fatty acid methyl ester (FAME)-trimethylsilyl (TMS) derivatives of lipids extracted from each spot. (**b**) MS profiles of FAME-TMS derivatives of ricinoleic acid (RA) detected at 11 min from spot Nos. 1–3, respectively. EPA, eicosapentaenoic acid; IS, internal standard; RA-TMS, trimethylsilyl ester of ricinoleic acid.

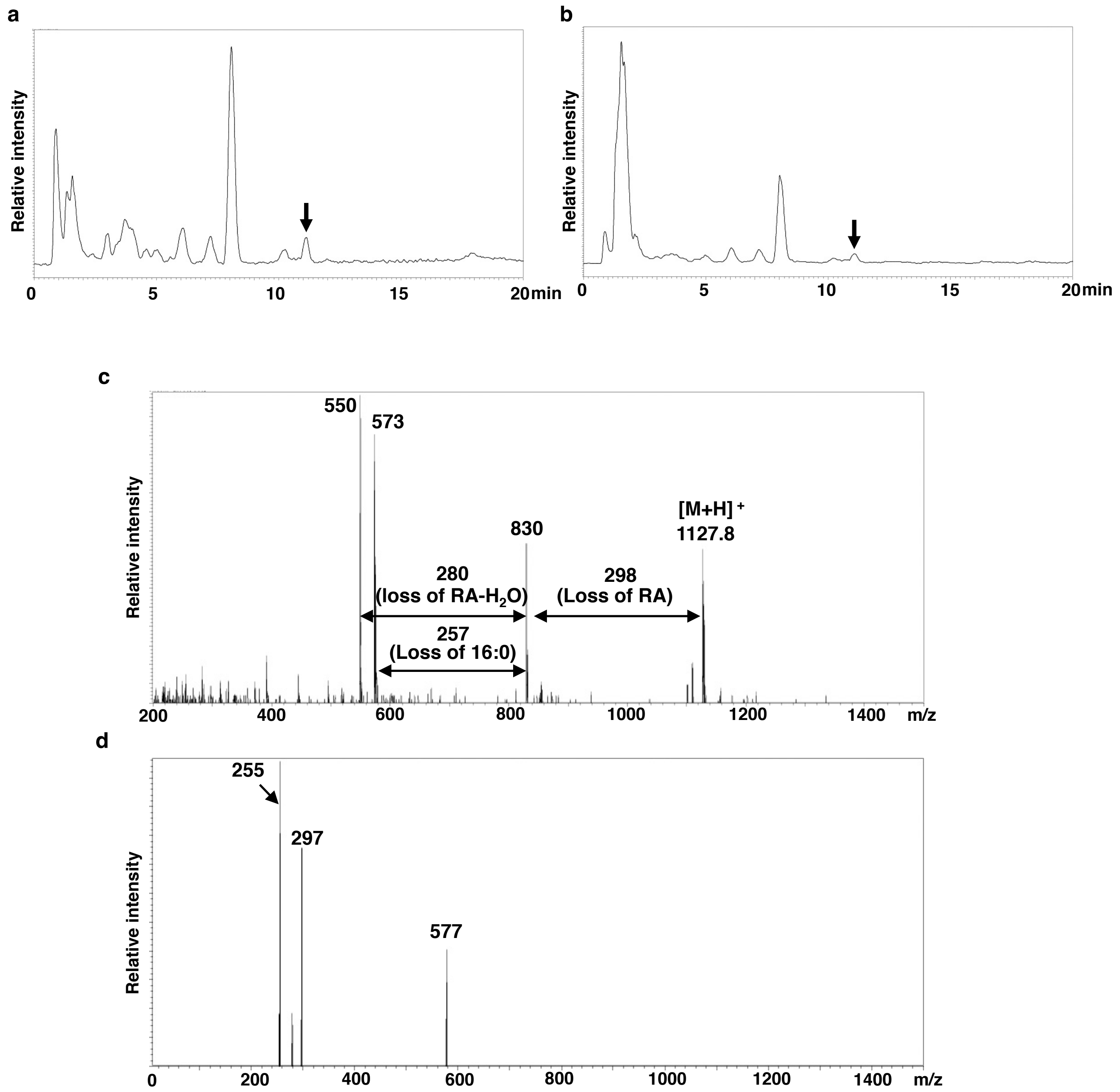

Supplementary Fig. S9: Liquid chromatography coupled with tandem mass spectrometry (LC-MS/MS) analysis of spot No. 2 containing ricinoleic acid (RA) in Cp4 cells shown in Fig. 3. Total ion chromatography in atmospheric pressure chemical ionisation positive (**a**) and negative (**b**) modes. (**c**) Full scan profile of a peak at 11.1 min indicated by arrows in (**a**) and (**b**), in electrospray ionisation (ESI) positive mode by AutoMSMS measurement. The fragment masses and ratios are consistent with an RA-RA estolide structure and two 16:0 as each side-chain fatty acid in triacylglycerol (TAG). (**d**) Scan profile with +45 V fragmentor voltage in ESI negative mode. Three fragment ions at m/z = 255, 297, and 577 corresponding to 16:0, RA, and dehydrated RA-RA estolide, respectively, were detected.

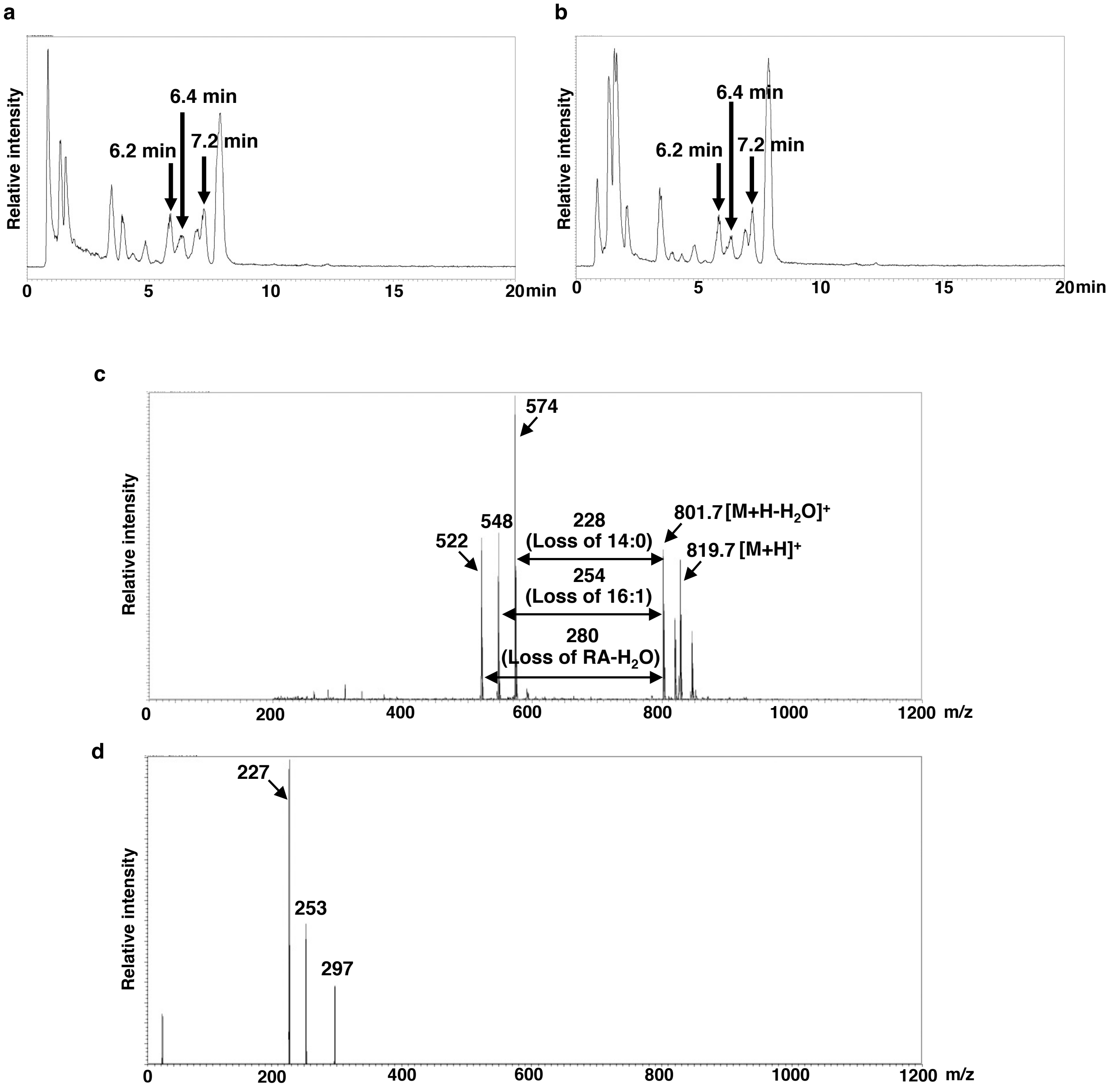

Supplementary Fig. S10: Liquid chromatography coupled with tandem mass spectrometry (LC-MS/MS) analysis of spot No. 3 containing ricinoleic acid (RA) in Cp4 cells shown in Fig. 3. Total ion chromatography in atmospheric pressure chemical ionisation in positive (**a**) and negative (**b**) modes. (**c**) Full scan profile of a peak at 6.2 min in (**a**) and (**b**), in electrospray ionisation (ESI) positive mode by AutoMSMS measurement. The fragment masses and ratios are consistent with a RA, a 14:0, and a 16:1 as each side-chain fatty acid in triacylglycerol (TAG). (**d**) Scan profile with +45 V fragmentor voltage in ESI negative mode. Three fragment ions at m/z = 227, 253, and 297 corresponding to 14:0, 16:1, and RA, respectively, were detected.

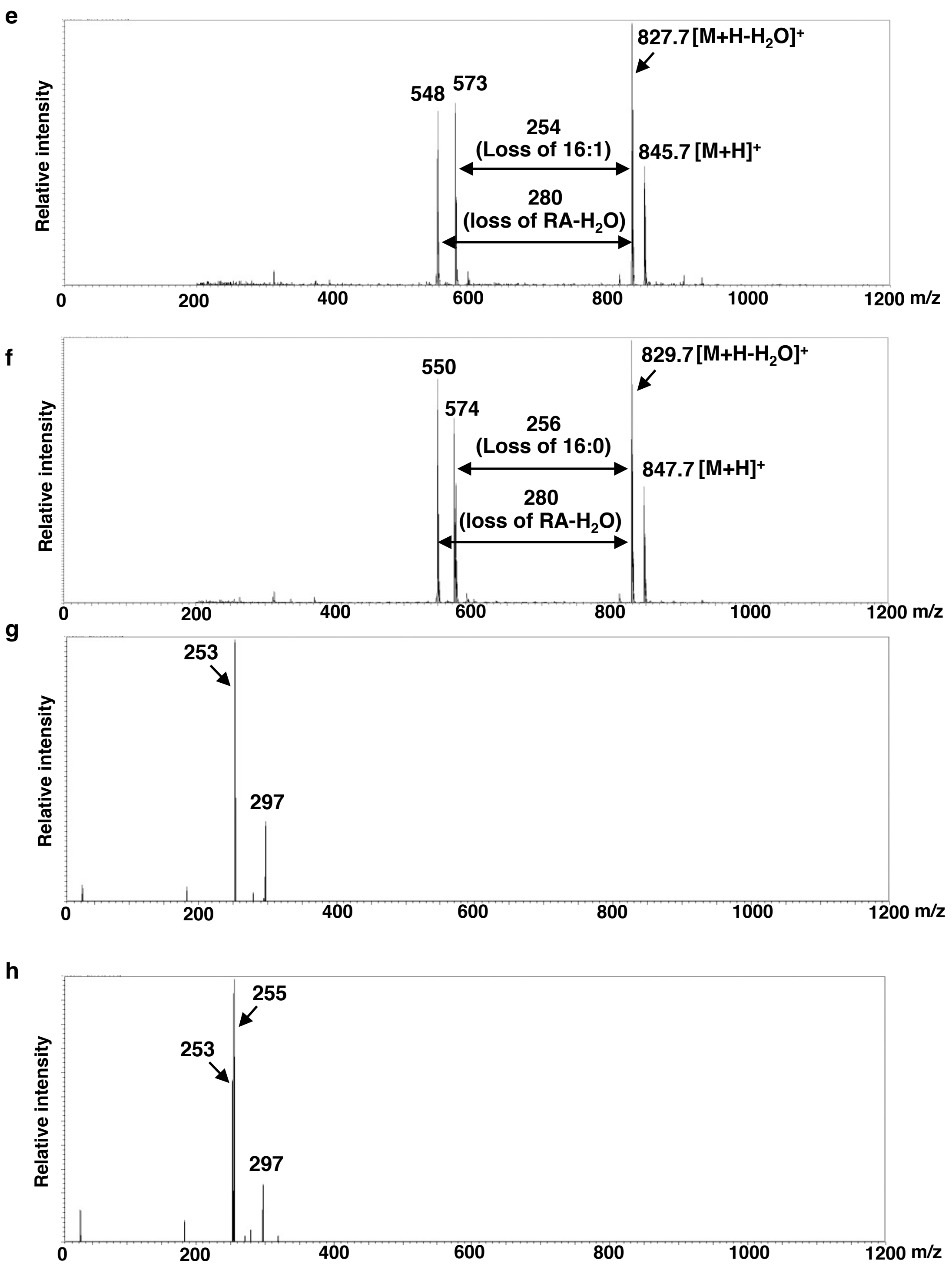

Supplementary Fig. S10 (continued): Full scan profile of a peak at 6.4 min (**e**) and 7.2 min (**f**) in ESI positive mode. The fragment masses and ratios were consistent with a RA, and two 16:1 for the peak at 6.4 min, and a RA, a 16:0 and a 16:1 for the peak at 7.2 min as each side-chain fatty acid in TAG. Scan profile of the peak at 6.4 min (**g**) and 7.2 min (**h**) with +45 V fragmentor voltage in ESI negative mode. Two fragment ions at m/z = 253 and 297 corresponding to 16:1 and RA were detected in the peak at 6.4 min in (**g**). Three fragment ions at m/z = 253, 255, and 297 corresponding to 16:1, 16:0, and RA were detected in the peak at 7.2 min in (**h**).

a

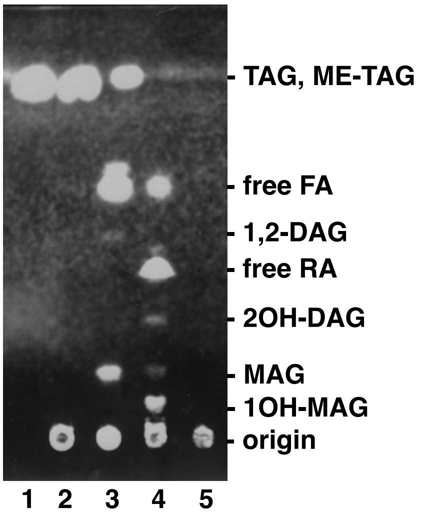

b

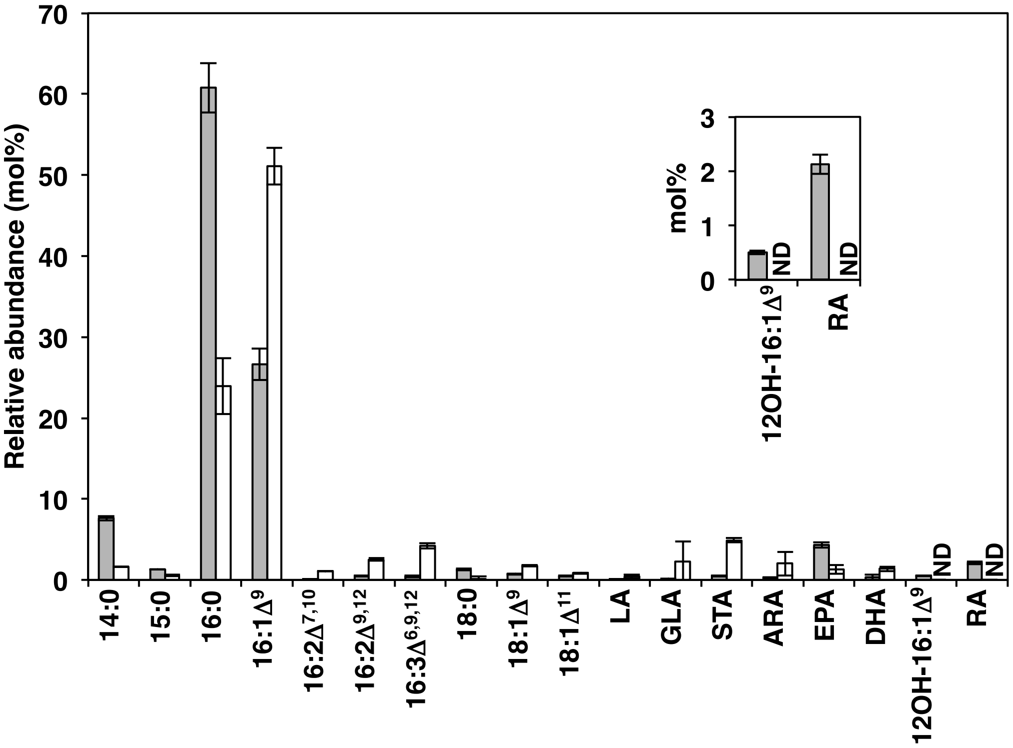

Supplementary Fig. S11: Positional analysis of monoestolide (ME) triacylglycerol (TAG) in Cp4 cells. (**a**) Thin layer chromatography (TLC) of purified TAG from Cp4 cells hydrolysed by *R. arrhizus* lipase. Lane 1, purified TAG. Lane 2, purified TAG incubated with reaction buffer. Lane 3, purified TAG incubated with lipase and reaction buffer. Lane 4, castor oil incubated with lipase and reaction buffer. Lane 5, mixture of lipase and reaction buffer. (**b**) Fatty acid composition of free fatty acids (grey bars) derived from the *sn-1*/*sn-3* position of the glyceryl backbone of TAG, and residual monoacylglycerol (MAG; open bars) containing *sn-2* position acyl groups. ARA, arachidonic acid; DHA, docosahexaenoic acid; EPA, eicosapentaenoic acid; GLA, γ-linolenic acid, LA, linoleic acid; RA, ricinoleic acid; STA, stearidonic acid. ND, not detected.

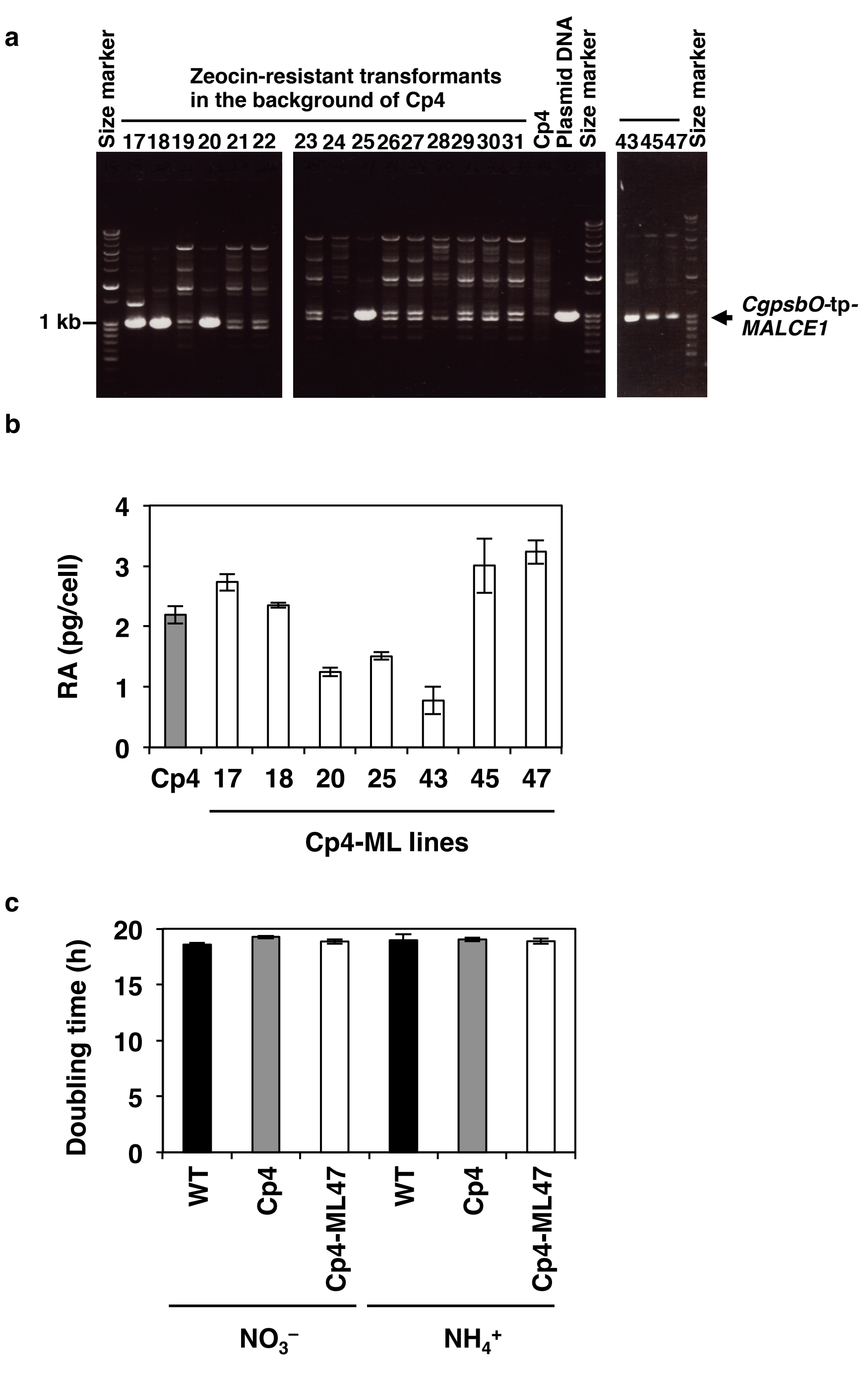

Supplementary Fig. S12: Screening of transgenic lines co-expressing *CpFAH* and *MALCE1* genes. (**a**) Genomic-PCR of transgenic lines containing a *MALCE1*-expression plasmid introduced into Cp4 cells. Representative results of genomic PCR for Zeocin-resistant clones No. 17–31, 43, 45, and 47 are shown. Size marker, 1-kb DNA size marker (Thermo Fischer Scientific); Cp4, genomic DNA of the Cp4 line (negative control); plasmid DNA, the expression plasmid DNA used for transformation (positive control). (**b**) Amount of ricinoleic acid (RA) in the parental Cp4 and seven transgenic lines harbouring both *CpFAH* and *MALCE1* expression plasmids cultured for 7 d at 15ºC in normal Daigo's IMK medium. (**c**) Growth curve of wild-type (WT), Cp4, and Cp4-ML47 lines for 7 d at 15ºC in normal Daigo's IMK medium containing NO_3^- or modified Daigo's IMK medium containing NH_4^+. Data in all experiments indicate mean value ± SD from three biological replicates.

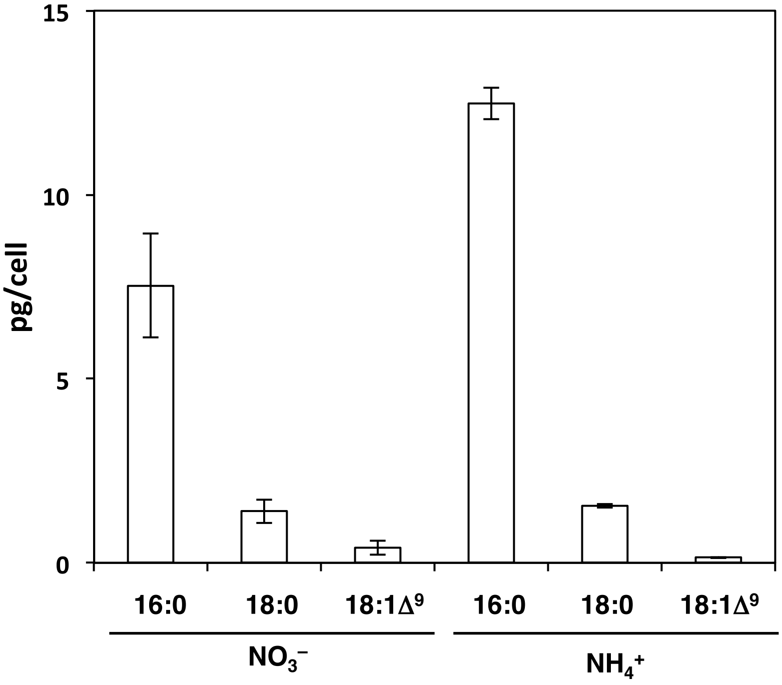

Supplementary Fig. S13: Contents of 16:0, 18:0, and 18:1Δ^9 fatty acids in Cp4-ML47 cells cultured at 15ºC for 7 d in normal Daigo's IMK medium containing NO_3^- or modified Daigo's IMK medium containing NH_4^+.

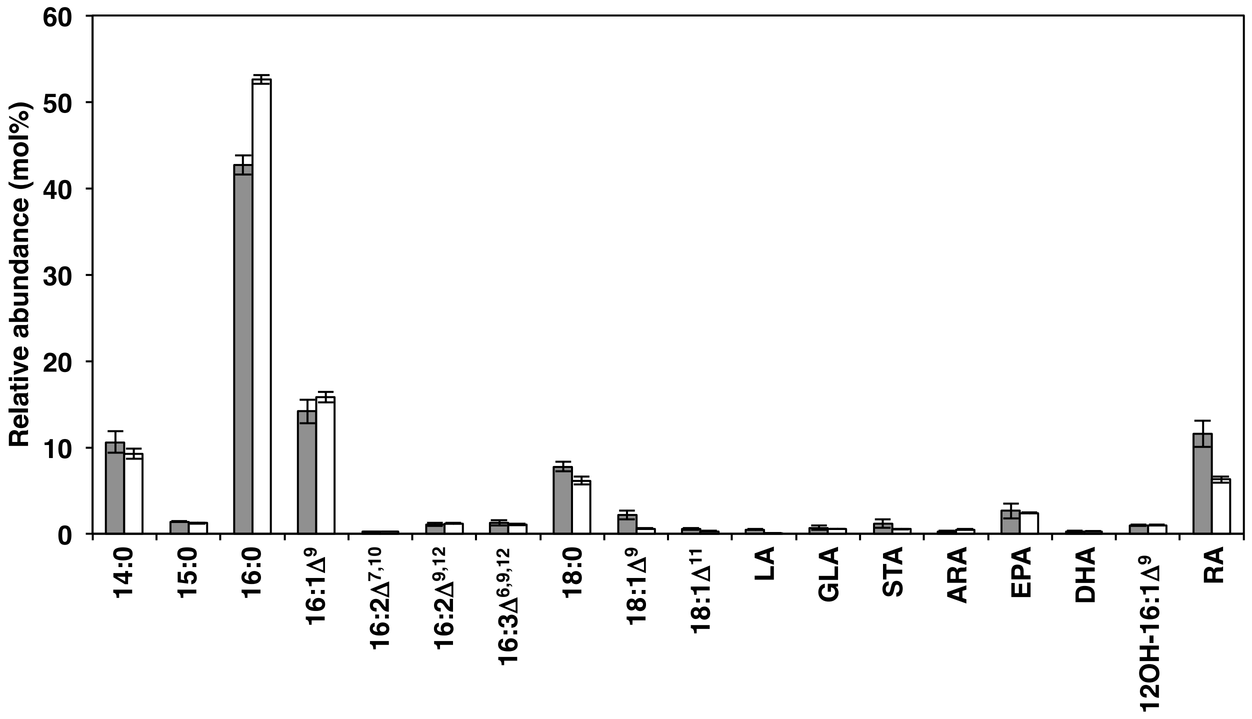

Supplementary Fig. S14: Fatty acid compositions of total lipids in Cp4-ML47 cells cultured at 15ºC for 7 d in normal Daigo's IMK medium containing NO_3^- (grey bars) or modified Daigo's IMK medium containing NH_4^+ (open bars). ARA, arachidonic acid; DHA, docosahexaenoic acid; EPA, eicosapentaenoic acid; GLA, γ-linolenic acid, LA, linoleic acid; RA, ricinoleic acid; STA, stearidonic acid.

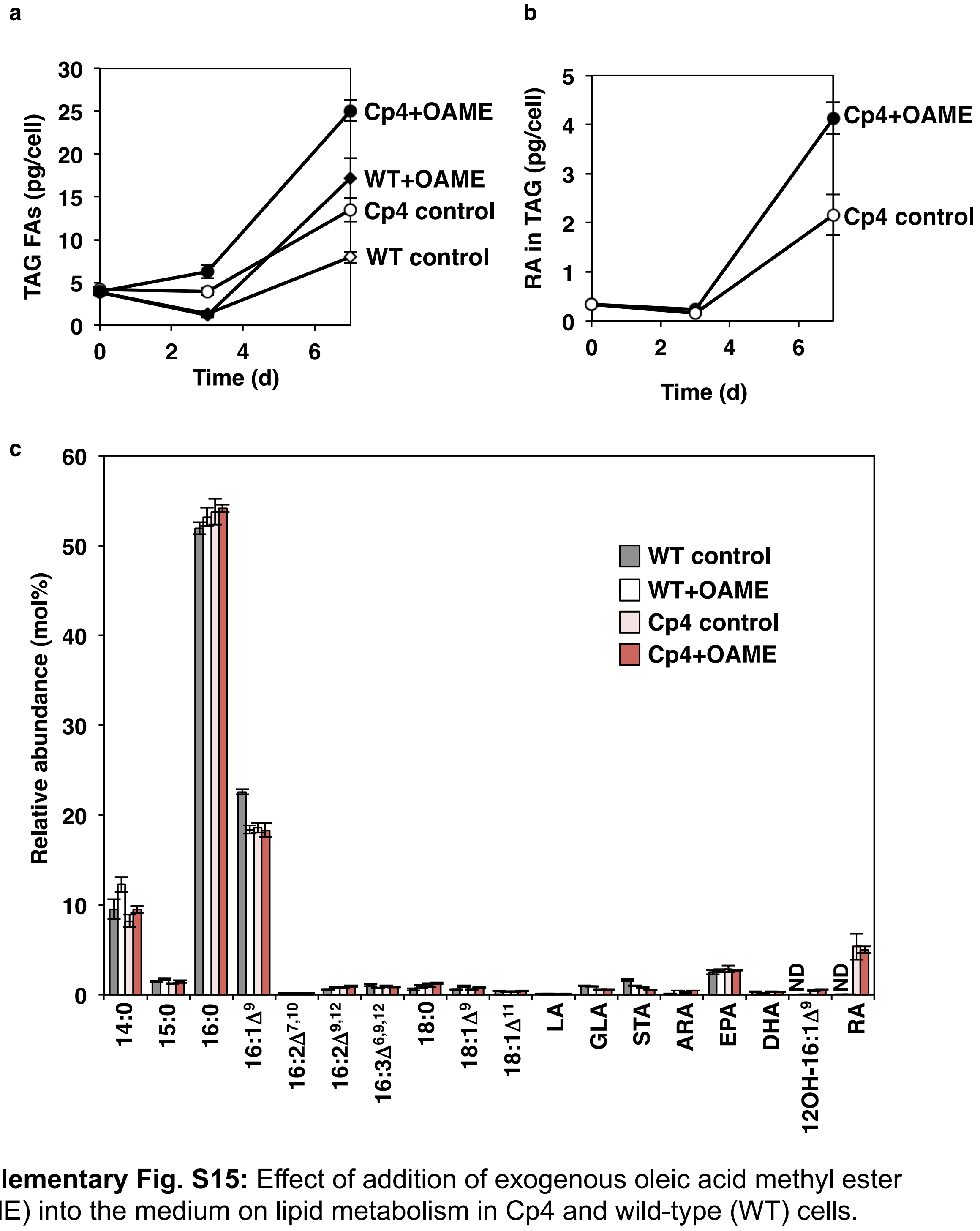

Supplementary Fig. S15: Effect of addition of exogenous oleic acid methyl ester (OAME) into the medium on lipid metabolism in Cp4 and wild-type (WT) cells. Changes of triacylglycerol (TAG) content (**a**) and ricinoleic acid (RA) content in TAG (**b**) per cell cultured at 15ºC supplemented with OAME resolved in ethanol at final concentration 4 µg/ml, or ethanol only as a control. (**c**) Fatty acid composition of TAG in WT and Cp4 cells cultured for 7 d. ARA, arachidonic acid; DHA, docosahexaenoic acid; EPA, eicosapentaenoic acid; GLA, γ-linolenic acid, LA, linoleic acid; RA, ricinoleic acid; STA, stearidonic acid. ND, not detected.

Supplementary Table S1: Primer sequences.

**For construction of *CpFAH* expression plasmid in yeast**		
	Forward (5’-3’)	Reverse (5’-3’)
	CTGTATAAGCTTATGGCTTCCGCTACTC	CTGCCTTCTAGACTACTGAGTCTTCATTG
**For genomic PCR screening of transformants harbouring pCgLhcr5p-*CpFAH* and pCgNRp/*CgpsbO*-tp-*MALCE1***		
	Forward (5’-3’)	Reverse (5’-3’)
pCgLhcr5p-*CpFAH*	TGGGTGAACCACTGGCTCGTTGC	TGCATGCATGCACTACTGAGTCTTC
pCgNRp/*CgpsbO*-tp-*MALCE*	CTGCAGATGAAGCTCGCTCTTGCATC	TCTAGATTACTGAGCCTTTTTCTGCGCG
**For construction of *CpFAH* and MALCE1 expression plasmids**		
	(5’-3’)	
*CpFAH-Bgl*II*-fw*	GGAAGATCTTCCATGGCTTCCGCTA	
*CpFAH-Nsi*I*-rv*	TGCATGCATGCACTACTGAGTCTTC	
InFusion_NRp/CgpsbOtp_fw	TTTATAAAGCGGATCCATGAAGCTCGCTCTTGCATC	
*PsbOsignal_Afl*II*_rv*	GTCGACTCTAGACTTAAGAGCAGCCTTTCC	
*MALCEI_Afl*II*_fw*	CTTAAGATGGAGTCTGGACCAATGCCTG	
*MALCEI_Xba*I*_rv*	TCTAGATTACTGAGCCTTTTTCTG CGCG	
*Sh_ble_Bgl*II*_fw*	TCTAGATCTATGGCCAAGTTGACCAGTGCC	
*Sh_ble_Nsi*I*_rv*	TATATGCATTCAGTCCTGCTCCTCGGCCAC	
**For qRT-PCR**		
	Forward (5’-3’)	Reverse (5’-3’)
CpFAH	CTATTTTCGAAGCCCGACAG	CCAGTGGTTCACCCAGAGAT
MALCE1	CATGTTCCCAGCAATGGTCAAG	GTAGCCTAGGGCGTTGTTCCAC
CgLhcr5	ATGCCAGGAGATTATGGATTTG	AAATTCAGATAGAGGCCAACCA
CgNR	CATTCTTTCCCACGATTCTTTTATG	ATTTGACGAAATTGGAGTGTATCG
Cg α*-tubulin*	GCTTTATCATCCCGAGCAAA	GTCGCATGGAACACAAGAAA
